# An Artificially Selected Cytokinin‐Pathway Transcription Factor Balances Soybean Yield and Pathogen Resistance

**DOI:** 10.1002/advs.76615

**Published:** 2026-07-20

**Authors:** Qun Ma, Yaqing Chen, Yiwen Gao, Xueting Hu, Qun Yang, Yucheng Liu, Peiyong Xin, Jijun Yan, Jinfang Chu, Lei Li, Zhixi Tian, Bo Ren

**Affiliations:** ^1^ Laboratory of Advanced Breeding Technologies Institute of Genetics and Developmental Biology Chinese Academy of Sciences Beijing China; ^2^ Institute of Genetics and Developmental Biology Chinese Academy of Sciences Beijing China; ^3^ University of Chinese Academy of Sciences Beijing China; ^4^ Key Laboratory of Seed Innovation National Center for Plant Gene Research (Beijing) Institute of Genetics and Developmental Biology Chinese Academy of Sciences Beijing China; ^5^ Yazhouwan National Laboratory Sanya Hainan China

**Keywords:** artificial selection, crop domestication, cytokinin signaling, *Glycine max*, growth–defense trade‐off, soybean yield

## Abstract

Soybean contributes to sustainable food and feed production, but some modern varieties favor yield at the cost of disease resistance. Using a bottom‐up approach, we demonstrate that the RR2b transcription factor activates cytokinin signaling for plant growth and reproductive fitness but represses disease‐resistance and root‐nodulation pathways. *RR2b* was artificially selected during domestication and improvement, and its expression level correlate with ATT repeat polymorphisms in its promoter. Most wild soybeans and landraces have either more or less ATT repeats: more repeats lead to weak *RR2b* expression with lower yield and enhanced resistance to soybean bacterial blight, and fewer repeats cause strong *RR2b* expression, higher yield, but reduced blight resistance. Elite cultivars instead balance yield and defense via moderate *RR2b* expression and several ATT repeats. This confers contrasting RR2b functionality across haplotypes in response to contrasting selection pressures and offers insights into decoupling trade‐offs between yield and blight resistance to enhance crop productivity.

## Graphical abstract

A cytokinin pathway transcription factor, RR2b, was artificially selected during soybean domestication and improvement based on its differential transcriptional activity, which correlates with ATT repeat polymorphisms in its promoter. RR2b balances yield and defense by fine‐tuning its expression level and offers a promising target for decoupling trade‐offs between yield and resistance to enhance crop productivity.

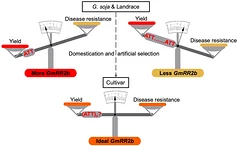

## 1 Introduction

Crop domestication is considered one of the most significant milestones in human history, marking the transition of human society from a hunting‐gathering lifestyle to an agricultural‐production system. By around 4000 years ago, humans had successfully domesticated all major crops that are essential to our diets today, including the legume cultivated soybean *Glycine max*, from its wild progenitor *G. soja* [1]. Today, global soybean production reaches approximately 400 million tons per year, accounting for about half of the world's oilseed production and contributing over a quarter of the world's feed protein [2]. Soybean fixes atmospheric nitrogen into plant‐available nitrate through symbiotic nitrogen fixation, with the potential to fix 15–20 million tons of nitrogen in the soil each year [3]. This dramatically reduces the economic and environmental costs of crop cultivation and enables sustainable agriculture. The soybean industry suffers annual losses exceeding $US25 billion due to pests and diseases. During soybean domestication, a series of favorable traits, such as yield, seed size, seed hardness, non‐shattering, and oil content, were artificially selected to meet human needs. However, this also led to a decline in the genetic diversity of soybean populations (i.e., a genetic bottleneck), making it difficult to breed high‐yield and disease‐resistant varieties [4].

Identification of adaptive genes controlling domestication‐related traits and characterizing their genetic and molecular mechanisms are integral to understanding the dynamic process of crop domestication and often serve as a launch pad for the delivery of improved elite cultivars. There are two principal approaches to identify adaptive genes. A top‐down approach (genetic mapping of domestication traits) starts with a phenotype of interest and then seeks to identify causal genomic regions by genetic analysis, such as a genome‐wide association study (GWAS) and linkage‐disequilibrium (LD) mapping. The other is a bottom‐up approach (genome scans for footprints of selection), which begins with identification of adaptation signals in a set of genes or a region in the genome using population‐genetic methods, and then moving from gene to phenotype by molecular methods [5]. To date, most genes isolated by the top‐down approach are so‐called ‘low‐hanging fruit’, which are single, large‐effect loci controlling major domestication traits, such as *fw2.2* for fruit size in tomato [6], *Sh4* for non‐shattering in rice [7], *tb1* for plant and inflorescence architecture in maize [8], *Q* for threshability in wheat [9], *GmHs1‐1* for hard‐seed in soybean [10], and *G* for dormancy in soybean, rice and tomato [11]. However, the number of genes identified through this method is rather limited. In contrast, a bottom‐up approach can identify a greater number of putative domestication loci, e.g., 55 loci in rice [12], 484 in maize [13], and 230 in soybean [14]. A major limitation of the bottom‐up approach is that resolving the functional consequences of the region/s of interest remains a complex experimental challenge.

As an essential plant growth hormone with crucial developmental functions, a broader understanding of how cytokinins regulate important agronomic traits makes this classic hormone an emerging genetic target for crop yield improvement [15, 16, 17, 18, 19, 20]. Cytokinin signaling in plant cells is transduced through a two‐component system, with B‐type response regulators (RRs) serving as positive transcription factors [21] that are involved in regulating soybean root growth, seed size, and phosphorus uptake [22, 23, 24]. Here, we used a bottom‐up approach to show that a locus containing the transcription‐factor gene *RR2b* in the soybean cytokinin signaling pathway is situated among several pathogen‐resistance genes subject to a selective sweep. We demonstrated that RR2b is a cytokinin‐pathway activator and a repressor of root nodulation and blight resistance, and differential repeats of an ATT insertion element in the *RR2b* promoter region alters its expression levels. Mechanism analysis revealed that RR2b regulates soybean blight resistance by controlling reactive oxygen species (ROS) generation, and balances soybean yield and blight resistance through the precise regulation of its transcription levels. This study provides a new insight into how fine‐tuning transcription factor expression orchestrates yield and disease resistance in crop breeding.

## 2 Results

### 2.1 RR2b was Strongly Selected During Soybean Domestication for its Transcriptional Activity

Because cytokinin has major impacts on several important soybean traits, including seed size, nutrient use, root architecture and root nodulation, we assessed whether components of the cytokinin pathway (Table S1) were selected during soybean domestication and subsequent improvement breeding, by plotting nucleotide diversity (π) and divergence index (*F*_st_). Among all 147 annotated genes involved in cytokinin biosynthesis, transport and signal transduction in soybean, only one, *RR2b* (*Glyma.15G145200*), which encodes a type‐B response regulator transcription factor, is located in a genomic region subjected to both a domestication and improvement selective sweep (Figure 1a, top). This hints at the importance of this region for agronomically desirable traits and soybean performance, and is consistent with a previous observation of genes related to domestication and improvement [14]. In this selection‐sweep region, there are 10 annotated disease‐resistance or drought‐induced genes, implying that *RR2b* was potentially co‐selected along with these resistance‐related genes (Figure S1a). *RR2b* is also located in certain reported disease‐resistance‐related QTLs, including for *Phytophthora sojae* (the causal pathogen of stem and root rot), *Fusarium solani* (sudden‐death syndrome), *Phakopsora pachyrhizi* (soybean rust), *Heterodera glycines* (nematode), and isoflavone‐metabolism genes, including genistein, glycitein, and isoflavone (Figure S1b), all of which contribute to defenses against biotic and abiotic stress. Interestingly, no polymorphism was found in the coding sequence of *RR2b* in these published core‐soybean germplasm [14], whereas an InDel of varying (ATT) repeats was found in its promoter at the –100 bp position (Figure 1a, bottom). Based on the number of these (ATT) repeats, we classified the germplasm population [14] into three haplotypes: haplotype 1 (HT1) containing 15 or 16 (ATT) repeats, haplotype 2 (HT2) containing fewer than 14 (ATT) repeats, and haplotype 3 (HT3) containing 17 or more (ATT) repeats (Table S2). A survey of the core soybean population comprising 296 sequenced accessions found that all three haplotypes have comparable frequencies in wild soybean *G. soja* (36%, 28%, and 36%, respectively). In landraces, the portion of HT1 increased to 79%, with HT2 and HT3 reduced to 3% and 18%, respectively. In *G. max* cultivars, HT1 frequency is predominant (90%) over HT2 (1%) and HT3 (9%) (Figure 1b), indicating that HT1 is the elite haplotype that is retained in modern soybean cultivars. These observations affirm that the genomic region in which *RR2b* is located was selected during both soybean domestication and its subsequent improvement breeding.

**FIGURE 1:**
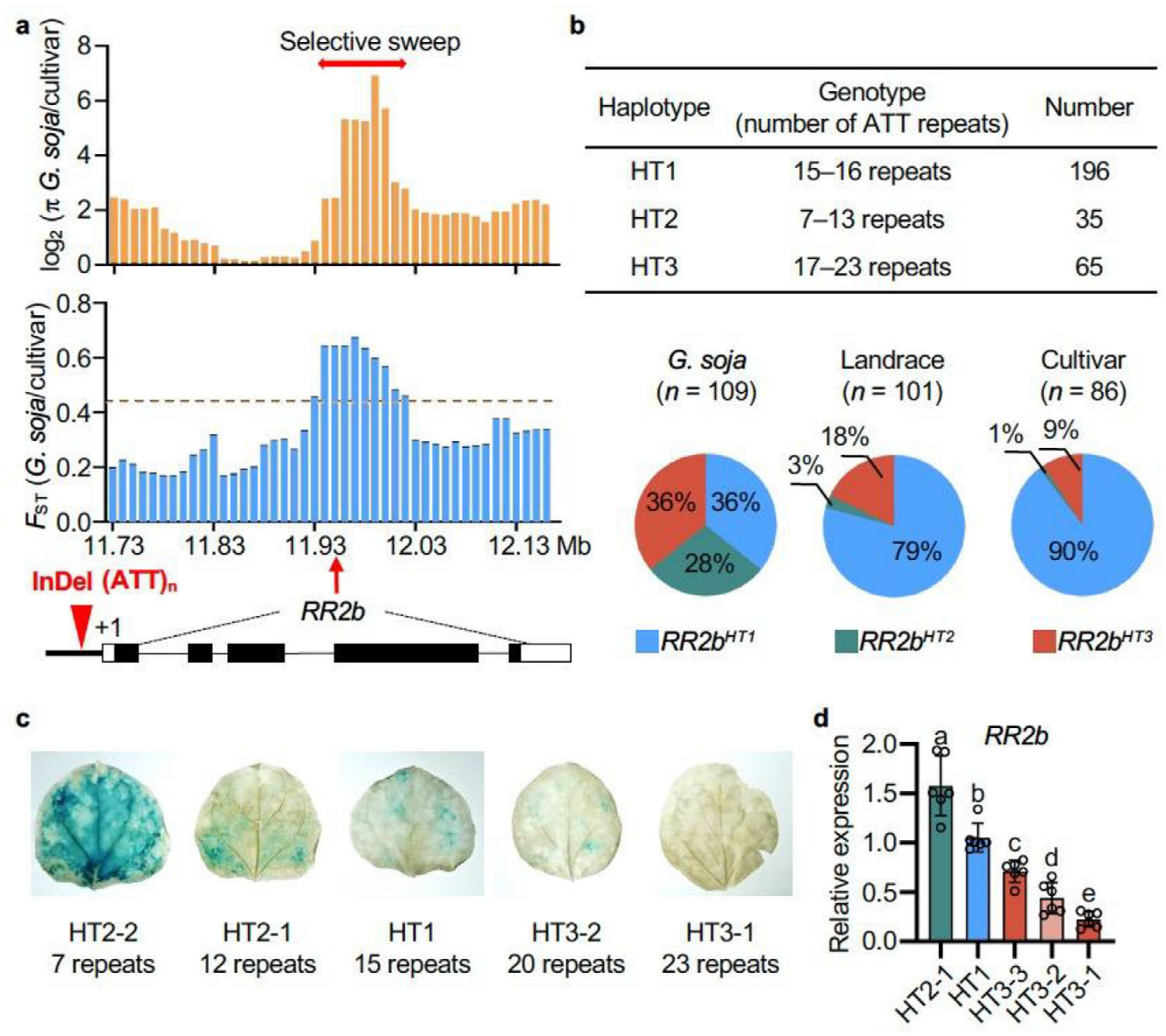
*RR2b* was selected during soybean domestication based on its transcriptional activity. (a) Nucleotide diversity (π, top) and population‐genetic differentiation (*F*_ST_, middle) at the *RR2b* locus. The double‐ended red arrow indicates the selective sweep, and the dashed orange line denotes the *F*_ST_ threshold. Bottom: *RR2b* genomic structure and its position on chromosome 15 in the analyses above. (b) Distribution of three main *RR2b* haplotypes (HT) in the core collection of 296 sequenced soybean accessions. (c) Histochemical analysis of GUS expression reporting the activity of different *RR2b* promoters containing various (ATT) insertion repeats in transiently transgenic *Nicotiana benthamiana* leaves. (d) *RR2b* expression levels in the roots of different soybean haplotypes. HT3‐3 contains 17 ATT insertion repeats in the promoter of *RR2b*. Relative expression levels were normalized to *ELF1b*. Data are presented as means ± SD from three biological replicates. At least six accessions were analyzed for each haplotype. Different letters indicate statistically significant differences at *p* < 0.05 by one‐way ANOVA with multiple comparison and Tukey's test.

Next, we tested whether the repeat insertions in the *RR2b* promoter affect its activity. Different *RR2b* promoter haplotypes fused to a *GUS* reporter (*RR2b_pro_:GUS*) were transformed into *Nicotiana benthamiana* leaves for transient expression. Activity of the *RR2b* promoter negatively correlated with the length of the insertion (Figure 1c). In soybean germplasm harboring different haplotypes, HT2 (containing short repeats in the promoter) has the highest *RR2b* expression level compared to that of HT1 and HT3 with longer repeats (Figure 1d).

In sequenced natural soybean populations, the minimal repeats of the ATT insertion is seven (Figure 1b). We generated an artificial *RR2b* promoter containing no repeats (designated ‘HT‐NR’) and tested its activity using transient dual‐luciferase assays, along with the natural haplotypes. This confirmed that *RR2b* promoter activity is negatively correlated with the insertion‐repeat number, that is, HT‐NR had the highest transcriptional activity, and HT3‐1 (with 23 insertion repeats, the most repeats in soybean germplasms we tested) had the lowest (Figure S2a,b). We infer that *RR2b* was selected during soybean domestication based on its differential transcriptional activity.

### 2.2 RR2b is a Transcriptional Activator of the Cytokinin Signal Transduction Pathway

*RR2b* is expressed in multiple organs in soybean (Figure S3a), and its protein product can activate the marker genes *RR5a* and *RR9c* [25] in the cytokinin signaling pathway (Figure S3b,c). As a typical transcription factor in this pathway, RR2b is composed of a receiver domain with conserved DDK residues, and an activation domain (Figure S3d). In Arabidopsis, the DDK domain of type‐B response regulators contains phosphorylation sites and a nuclear‐localization signal, and the ΔDDK–domain mutant can activate cytokinin signaling more efficiently [26]. Likewise, full–length soybean RR2b–GFP localized in the nucleus, whereas the single– and double–Asp deletion mutants failed to localize there and instead appeared in the cytosol and plasma membrane (Figure S3e). To investigate the relationship between RR2b and other type‐B RRs, we constructed a phylogenetic tree that includes all type‐B RR from Arabidopsis, soybean, and two other legume, *Medicago truncatula* and *Lotus Japonicus*. The four soybean RR2 cluster in one clade (bootstrap value = 100) together with Arabidopsis ARR1, ARR2, and their homologs in *M. truncatula* and *L. japonicus*, whereas they share low homology with other soybean type‐B RRs (bootstrap value = 58) (Figure S4). This suggests that the function of RR2s have diverged significantly compared to other type‐B RRs in soybean.

To understand the roles of RR2b in the soybean cytokinin pathway, we generated stable–transgenic plants over‐expressing *RR2b* fused to the 3xFLAG tag (OE), together with mutant alleles by gene editing (*rr2b*), all in the W82 wild‐type (WT) background (Figure S3f,g). Knockout and over‐expression of *RR2b* resulted in taller and shorter seedling heights, respectively (Figure 2a,b). We tested the physiological responses of these genotypes to exogenous supplementation with the cytokinin analog BAP (6‐benzylaminopurine). Less than 100 nm BAP did not inhibit the primary root length of WT plants. Along with higher BAP concentrations, WT root length was reduced, the *rr2b*‐*1* mutant showed reduced sensitivity, and *RR2b* OE plants were hypersensitive (Figure 2c). This supports that *RR2b* is a positive regulator of the cytokinin pathway in soybean, akin to the Arabidopsis homolog.

**FIGURE 2:**
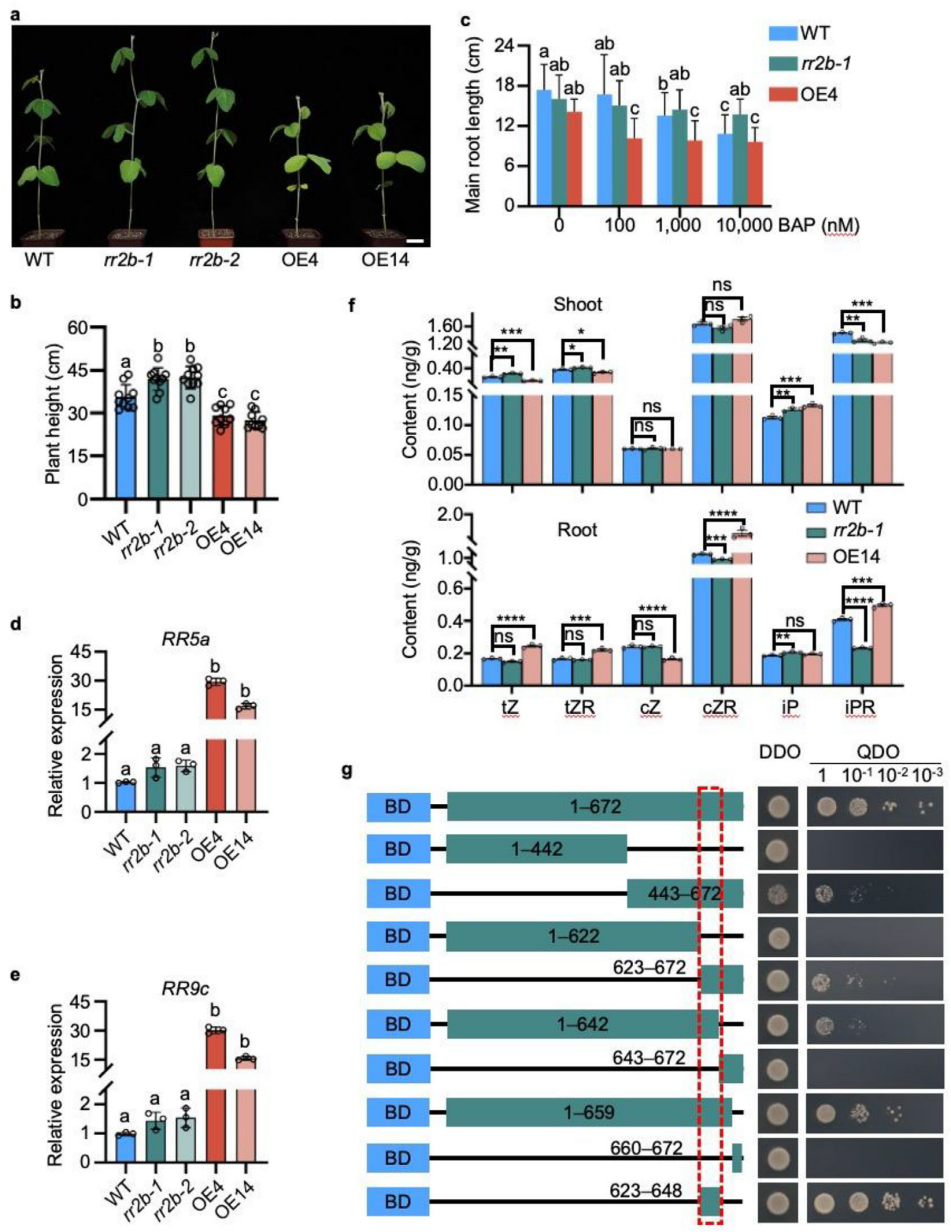
*RR2b* is a positive transcriptional regulator of the cytokinin‐signaling pathway. (a) Representative phenotypes of 21‐d‐old wild‐type c.v. W82, *rr2b* knockout and *RR2b* over‐expression (OE) plants. (b) Plant height for 21‐d‐old W82, *rr2b* knockout, and *RR2b* OE plants. Data are the means ± SD (n ≥ 15 individual plants). Three independent experiments were performed with similar results. (c) Primary root length for W82, *rr2b* knockout and *RR2b* OE transgenic lines in response to varying concentrations of exogenous BAP or mock treatment for 15 d. Data are means ± SD (n ≥ 13 individual plants). Three independent experiments were repeated with similar results. (d, e) Relative expression of *RR5a* (d) and *RR9c* (e) in W82, *rr2b* knockout, and *RR2b* OE transgenic lines. Data are means ± SD from three biological replicates. (f) Cytokinin contents in W82, *rr2b*‐1, and OE14 lines. Shoots and roots were harvested separately at 10 d and ground into powder. Trans‐Zeatin (tZ), trans‐zeatin riboside (tZR), cis‐zeatin (cZ), cis‐zeatin riboside (cZR), isopentenyladenine (iP), and isopentenyladenine riboside (iPR) were detected and quantified in these samples. Data are presented as means ± SEM (n = 3). Different numbers of asterisks indicate statistically significant differences relative to the WT control within each group, as determined by one‐way ANOVA test: ^****^ *p* < 0.0001, ^***^ *p* < 0.001, ^**^ *p* < 0.01, ^*^ *p* < 0.05, ns, *p* > 0.05. (g) Transactivation activity of RR2b domains in yeast. Full‐length RR2b or the indicated truncations (in green) were fused in‐frame with the GAL4 DNA‐binding domain (BD, in blue) in the pGBKT7 vector, respectively. Colony growth in selective medium indicates the transactivation of RR2b. DDO, SD/–Trp/–Leu; QDO, SD/–Trp/–Leu/–His/–Ade. The dashed‐red rectangle indicates the region comprising amino acids 623–648 is sufficient and necessary for RR2b activity. Expression levels in d and e were normalized to *ELF1b*. In b–e, different letters indicate statistically significant differences at *p* < 0.05, as determined by multiple‐comparison testing by one‐way ANOVA analysis with Tukey's test. Scale bars = 2 cm in a.

We also examined the induction of cytokinin‐pathway marker genes (Figure 2d,e). *RR5a* and *RR9c* are dramatically induced in *RR2b* OE plants, but are comparable to WT in *rr2b*‐*1* and *rr2b*‐*2* mutants, probably due to the compensation or severe redundancy amongst the 24 annotated type‐A *RRs* in soybean (Table S1). Then, cytokinin contents of *RR2b* knockout mutants and OE plants were tested. There are two main active forms of cytokinin in plants, *trans*‐zeatin (tZ) and *N* ^6^‐(*Δ* ^2^‐isopentenyl) adenine (iP). The tZ content in *RR2b* OE plant is lower than that in WT in the shoot and higher in the root whereas *rr2b* mutant has a higher tZ level in the shoot. The iP contents in the shoot of *rr2b* mutant and the OE plant are both higher than WT, and in the roots, *rr2b* mutant is slightly higher than WT. The storage and inactive forms of cytokinin, including tZ‐riboside (tZR), iP‐riboside (iPR), *cis*‐Zeatin (cZ), and cZ‐riboside (cZR), varies in different tissues of the *rr2b* mutant and OE plant (Figure 2f), indicating the multiple roles of cytokinin in different tissues and the complexity of cytokinin negative regulation feedback [21]. To identify the RR2b domain/s required for its activity, the functionality of truncation mutants was assayed in yeast. The RR2b C‐terminal is more important for its transcription activity than the N‐terminal, consistent with that in Arabidopsis [27, 28]. Only 26 amino acids (residues 623–648) are sufficient and necessary for this transcriptional activity in yeast (Figure 2g). Together, these data confirmed that RR2b is a transcriptional activator of cytokinin signaling in soybean.

### 2.3 RR2b Acts as a Transcriptional Repressor of Soybean Disease‐Resistance Pathways to Dampen ROS Bursts

To further study the biological roles of the transcription factor RR2b, we performed a CUT & Tag with *RR2b‐3*x*FLAG* transgenic plants and identified a core motif (Figure 3a). This RR2b binding motif contains the sequence TTGGAAT, which differs from the classic GARP‐motif (G/A)GAT(T/C) of type‐B RRs [26], indicating RR2b might have a distinctive function in soybean. We analyzed transcriptomes of WT, *rr2b* knockout, and over‐expression plants by RNA‐seq. Differentially expressed genes (DEGs) between these genotypes are highly enriched in pathogen resistance‐related pathways, including cell wall biogenesis, phenylpropanoid biosynthesis, plant‐pathogen interaction, and flavonoid biosynthesis pathway, which are also involved in legume‐rhizobia interactions for root nodulation (Figure S5). Candidate RR2b‐3xFLAG target genes were filtered out by overlapping putative target sequences identified by CUT & Tag with the DEGs identified in *RR2b* OE14 plants vs. WT. From this, we identified 274 candidates, of which 174 genes show the expected reciprocal expression pattern in the *rr2b* knockout and OE plants (Figure 3b and Table S3). These genes are also significantly enriched in disease resistance‐related pathways, for example, three known pathogen resistance genes, *PPR40* (*PENTATRICOPEPTIDE REPEAT 40*) [29], *ATKH* (*K HOMOLOGY DOMAIN‐CONTAINING PROTEIN*) [30], and *LRR4* (*LEUCINE RICH REPEAT PROTEIN 4*) [31] are up‐regulated in *rr2b* mutants and down‐regulated in *RR2b* OE plants (Figure 3c–e), suggesting that RR2b suppresses the expression of these defense genes. We validated CUT & Tag peaks (Figure 3f–h) by electrophoretic mobility shift assay (EMSA). RR2b–GST directly binds to TTGGAAT *cis*‐elements located in the promoters of *PPR40*, *ATKH8* and *LRR4* (Figure 3i–k). This was validated *in planta* by dual‐luciferase reporter assays in which the activity of these promoters was reduced in the presence of RR2b, compared to empty‐vector (EV) controls (Figure 3l–n). Altogether, these results indicated that the transcription factor RR2b acts as a repressor of these soybean disease‐resistance genes.

**FIGURE 3:**
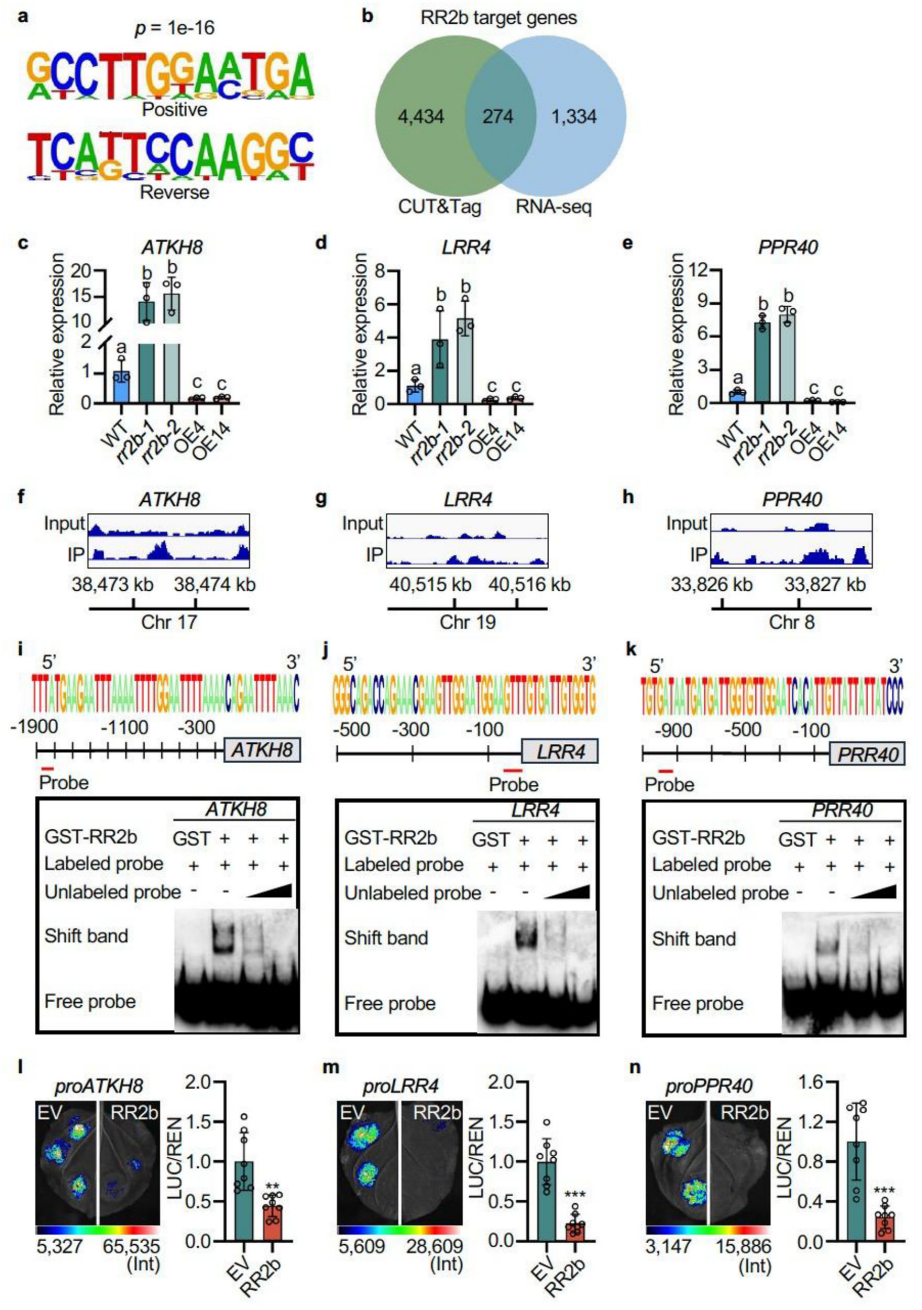
RR2b acts as a transcriptional repressor of soybean disease resistance. (a) HOMER motif analysis of regions enriched within RR2b binding regions experimentally determined by CUT & Tag. (b) Venn diagram of overlap between putative RR2b target genes identified by CUT & Tag and differentially expressed genes in *RR2b* OE14 relative to W82 as identified by RNA‐seq. (c–e) Relative expression of *ATKH8* (c), *LRR4* (d) and *PPR40* (e) in W82, *rr2b* knockout and *RR2b* OE transgenic lines. Expression levels were normalized to *ELF1b*, and data are means ± SD from three biological replicates. Different letters indicate statistically significant differences at *p* < 0.05 by one‐way ANOVA analysis with Tukey's test. (f–h) CUT & Tag analysis of RR2b–3xFLAG preferentially binding to the promoter of *ATKH8* (f), *LRR4* (g) and *PPR40* (h). (i–k) EMSA of GST–RR2b binding in vitro to *cis*‐elements within promoters of *ATKH8* (i), *LRR4* (j) and *PPR40* (k). The colored nucleotide sequence at the top of each panel represents the probe sequence. The red lines indicate the exact location of the probe within the promoter region. Three independent replicates were performed, and a representative result is shown. (l–n) Transient dual‐luciferase assays in *N. benthamiana* leaves of RR2b binding to the promoter of *ATKH8* (l), *LRR4* (m) and *PPR40* (n). Shown are relative ratios of the transcriptional activities conferred by RR2b expression to the empty–vector control. LUC/REN, ratio of firefly luciferase (LUC) to *Renilla* luciferase (REN) activity. Data are presented as means ± SD (n = 8). Three independent experiments were repeated with similar results. Asterisks indicate statistically significant differences relative to the empty‐vector (EV) control. Two‐sided Student's *t*‐test, ^**^ *p* < 0.01, ^***^ *p* < 0.001.

Because RR2b binds to the promoters of select disease‐resistance‐pathway genes to inhibit their expression, we assessed disease symptoms of WT, *rr2b* knockout, and *RR2b* OE plants. *rr2b* mutants show enhanced resistance to *Pseudomonas syringae pv. glycinea*, which causes soybean bacterial blight, and *RR2b* OE plants are hypersusceptible to this pathogen relative to WT (Figure 4a,b). Consistent with this, expression of pathogen‐resistance marker genes *PR2* and *PR5* are strongly induced in *rr2b* mutants and repressed in *RR2b* OE plants (Figure 4c,d). Production of flg22‐triggered reactive oxygen species (ROS), an early hallmark of the pattern‐triggered immunity (PTI) response, is dramatically higher in *rr2b*‐*1* than in WT, and the ROS burst in *RR2b* OE plants is reduced compared to WT (Figure 4e,f).

**FIGURE 4:**
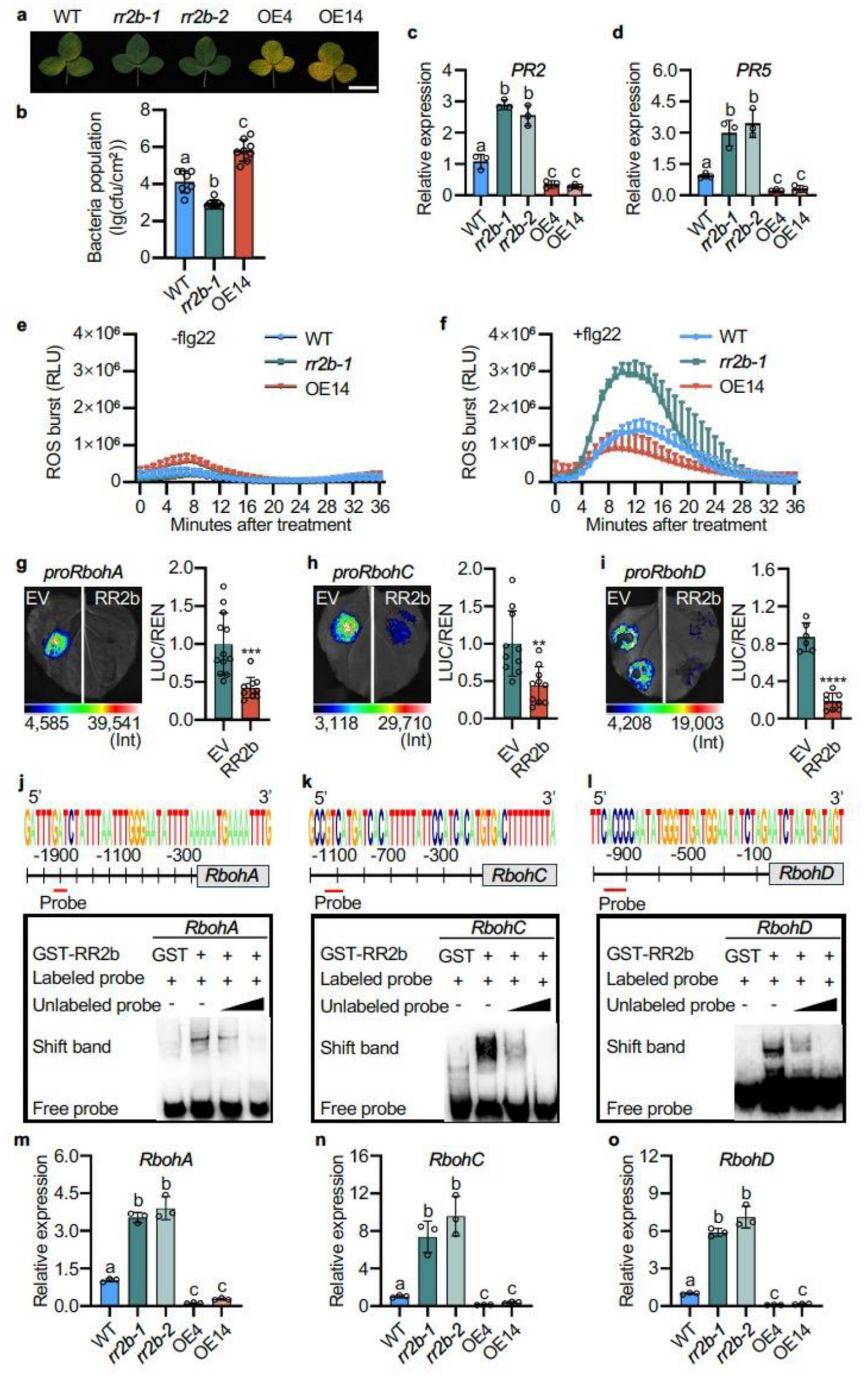
RR2b inhibits soybean disease resistance by dampening ROS bursts. (a, b) Representative images (a) and bacterial population (b) in leaves of W82, *rr2b* knockout, and *RR2b* OE lines inoculated with *Pseudomonas syringae* pv. *glycinea* (*Psg*). (a) Seven days after inoculation. Scale bars = 2 cm. (b) Population quantification. Data are means ± SD of *n* = 8. Three independent experiments were repeated with similar results. (c, d) Relative expression of *PR2* (c) and *PR5* (d) in W82, *rr2b* knockout, and *RR2b* OE transgenic lines. Data are means ± SD from three biological replicates. (e, f) ROS production induced by mock treatment (e) or flg22 treatment (f) in 4‐d‐old seedlings of W82, *rr2b* knockout, and *RR2b* OE lines. (g–i) Transient dual‐luciferase assays of RR2b binding to the promoter of *RbohA* (g), *RbohC* (h), *RbohD* (i). Shown are relative ratios of the transcriptional activities conferred by RR2b expression to the empty‐vector control. LUC/REN, ratio of firefly luciferase to *Renilla* luciferase activity. Data are means ± SD (n = 8). Three independent experiments were repeated with similar results. Asterisks indicate statistically significant differences relative to the EV control. Two–sided Student's *t*‐test, ^**^ *p* < 0.01, ^***^ *p* < 0.001, ^****^ *p* < 0.0001. (j–l) EMSA of GST–RR2b binding in vitro to *cis*‐elements in promoters of *RbohA* (j), *RbohC* (k) and *RbohD* (l). The colored nucleotide sequence at the top of each panel represents the probe sequence. The red lines indicate the exact location of the probe within the promoter region. At least three independent replicates are performed for each experiment, and a representative result is shown. (m–o) Relative expression of *RbohA* (m)*, RbohC* (n) and *RbohD* (o) in W82, *rr2b* knockout and *RR2b* OE lines. Expression levels in panels c, d, m–o were normalized to *ELF1b*, and data are presented as means ± SD from three biological replicates. In b–d and m–o, different letters indicate statistically significant differences at *p* < 0.05 by one‐way ANOVA analysis with Tukey's test.

We then examined the relationship between RR2b and RESPIRATORY BURST OXIDASE HOMOLOG (RBOH) enzymes, given that ROS is a well‐established signaling molecule in plant immunity, and this direct regulatory link provides a compelling mechanistic basis for the observed phenotype. The promoter activities of *RbohA*, *RbohC* and *RbohD* (Glyma.06G162300), which is also identified in the above overlapping analysis of CUT & Tag with DEGs (Table S3), were suppressed by RR2b in dual‐luciferase reporter assays (Figure 4g–i), and RR2b–GST could bind in vitro to sequences in these promoters in EMSAs (Figure 4j–l). Furthermore, *RbohA*, *RbohC* and *RbohD* expression levels are greatly increased in *rr2b* mutants and decreased in *RR2b* OE plants compared to WT (Figure 4m–o). These functional and genetic experiments indicate that RR2b represses expression of these *Rboh* genes at the transcriptional level, thereby dampening ROS bursts.

In Arabidopsis, ARR2 activates the defense marker gene *PR1* and various peroxidases [32, 33], which appears to be inconsistent with the findings of this study so far. Therefore, we simultaneously examined the effect of Arabidopsis ARR2 on soybean disease‐resistance‐pathway genes, as well as the regulatory role of soybean RR2b on Arabidopsis *PR1* and peroxidase genes. In dual‐reporter assays, Arabidopsis ARR2 inhibited the activity of soybean *ATKH8* and *LRR4* promoters (Figure S6a–c). Soybean RR2b was able to activate the Arabidopsis *PRX33* promotor but inhibited that of *PR1* promoter (Figure S6d–f). These results indicate that RR2b has species‐specificity in regulating plant disease resistance pathways and ROS production within its native plant context.

### 2.4 RR2b Balances Soybean Yield and Pathogen Resistance

As *RR2b* was selected during soybean domestication and improvement breeding, we examined the effects of *RR2b* haplotypes on agronomic traits under natural conditions in the field at three locations spanning from 37°26′ to 39°54′ of northern latitude. *rr2b* mutants were taller than WT and *RR2b* OE plants were shorter (Figure 5a and Figure S7a–c). By contrast, branch number (Figure S7d–f), total grain number per seedling (Figure S7g–i), pod number per plant, and the proportion of the pods containing one to four seeds (Figure S7j–l), are generally comparable across genotypes. *rr2b* mutants had lower yield, and *RR2b* OE plants had a higher yield than WT in 2024 (Figure 5b–d). Moreover, at these locations in 2023 and 2024, different *G. max* varieties with various insertion repeats in the *RR2b* promoter (with different expression levels) produced similar results, with the germplasm bearing shorter insertions having higher yield, and vice versa (Figure 5e–h). Analysis on seed weight per plants with a more diverse set of soybean germplasms (308 cultivars and 211 landraces, from the SoyOmics database) shows that HT1 accessions (containing fewer ATT repeats) has higher per‐plant yield than the HT3 accessions containing more ATT repeats (Figure S9a). These results support that *RR2b* expression levels are associated with soybean yield. For responses to *Pseudomonas syringae pv. glycinea* challenge, the more (ATT) repeats in the *RR2b* promoter, the stronger the ROS bursts and the fewer colonies (Figure 5i,j). These results collectively suggest that *RR2b* transcript level is positively related to soybean grain yield and negatively related to blight resistance.

**FIGURE 5:**
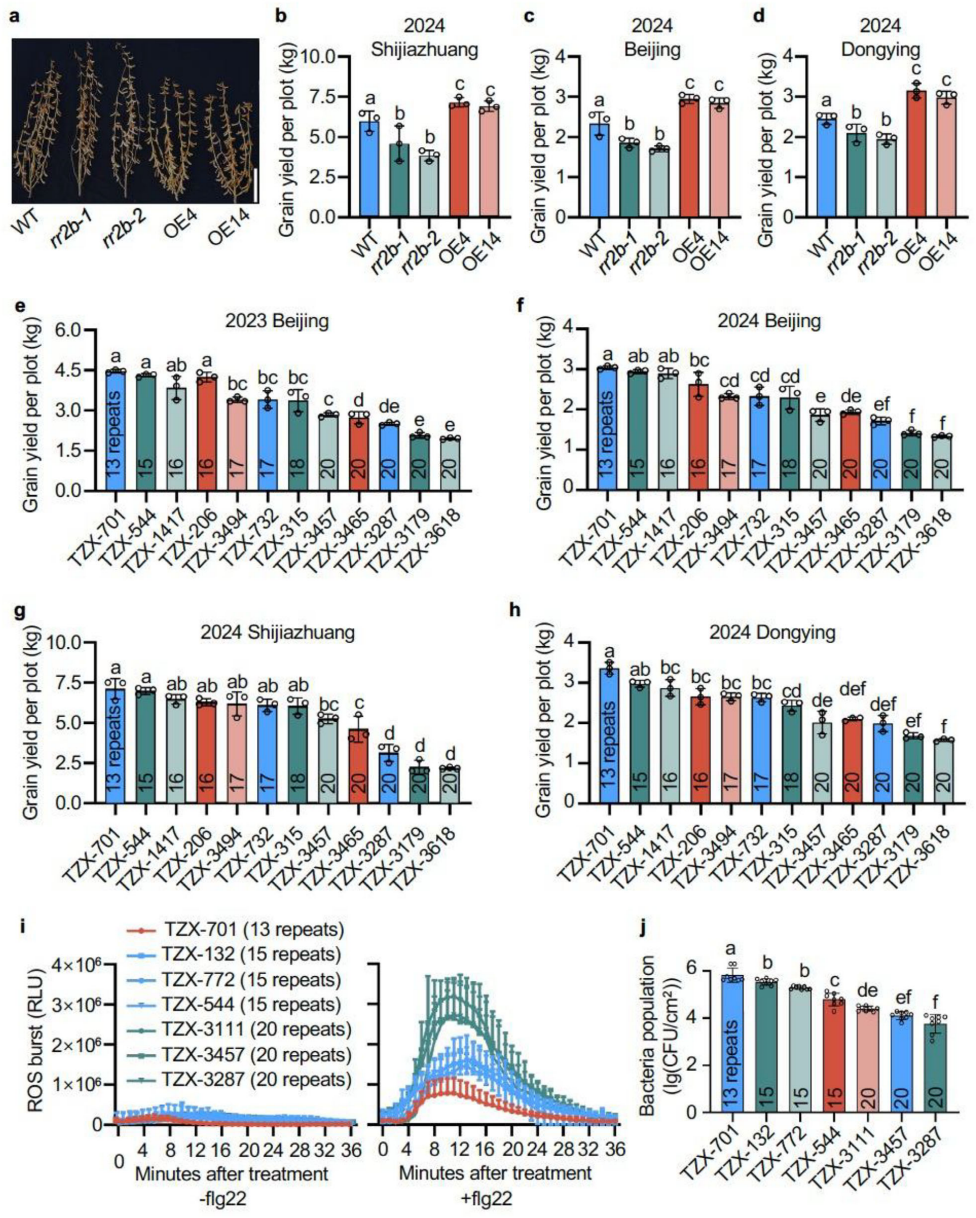
RR2b balances soybean yield and disease resistance. (a) Phenotype of W82, *rr2b* knockout and *RR2b* OE shoots at the harvest stage. Scale bar = 10 cm. (b–d) Quantification of grain yield per plot for W82, *rr2b* knockout and *RR2b* OE lines grown at Shijiazhuang (b), Beijing (c) and Dongying (d) field sites in 2024. (e–h) Quantification of grain yield per plot for soybean germplasm containing various insertion copies in the *RR2b* promoter grown at multiple field sites in 2023 and 2024. Three plots were grown for each line, with plot sizes in Shijiazhuang and Dongying of 15 m^2^ and in Beijing of 9 m^2^. (i) ROS production in 4‐d‐old HT1, HT2 and HT3 seedlings under mock treatment (left) or treated with flg22 (right). (j) Bacterial population in leaves of different soybean varieties inoculated with *Psg*. Data are means ± SD of *n* = 8. Data in (b–h, and j) are means ± SD, and different letters indicate statistically significant differences at *p* < 0.05 determined by one‐way ANOVA analysis with Tukey's test. For (e–j), the denoted repeats refer to the copy numbers of (ATT) insertions in the *RR2b* promoter.

Notably, one of the most important soybean domestication traits, seed size, as expressed by 100‐seed weight, was much lower in *rr2b* knockout plants than in WT, with *RR2b* OE plants consistently producing heavier seeds across all seasons and locations (Figure 6a–e). These results suggested that *RR2b* positively regulates soybean seed size. Indeed, *G. max* varieties, which contain landraces and elite cultivars, seed size positively correlates with *RR2b* transcript level in multiple field trials, with the high‐expression‐level *RR2b* germplasm containing fewer (ATT) insertion repeats having bigger seeds than the low‐expression‐level *RR2b* germplasm (Figure 6f–j). In *G. soja*, the seeds of which are generally smaller than those of landrace and cultivar, the number of (ATT) insertion repeats is also negatively correlated with the expression level of *RR2b*, and this appears to be unrelated to seed size (Figure S8a,b). Thus, we examined the expression levels of other key genes regulating seed size in different soybean germplasms to confirm their relationship with seed size. *GmSW17*, which positively regulates seed size [34], had statistically significant lower expression in *G. soja* than in landrace and cultivar, while *KIX8‐1*, which negatively regulates seed size [35], had statistically significant higher expression in *G. soja* compared to landrace and cultivar (Figure S8c,d). Furthermore, we selected a larger number of *G. max* varieties containing 211 landraces and 216 cultivars from the SoyOmics database for analysis of their hundred‐seed weights. In *G. max*, the hundred‐seed weight of HT1 accessions that contain fewer ATT insertion repeats was greater than that of HT3 accessions (Figure S9b), in agreement with our multiple field trials reporting hundred‐seed weight (Figure 6f–j). These findings suggest that *RR2b* may fine‐tune soybean seed size based on the major‐effect genes controlling seed size.

**FIGURE 6:**
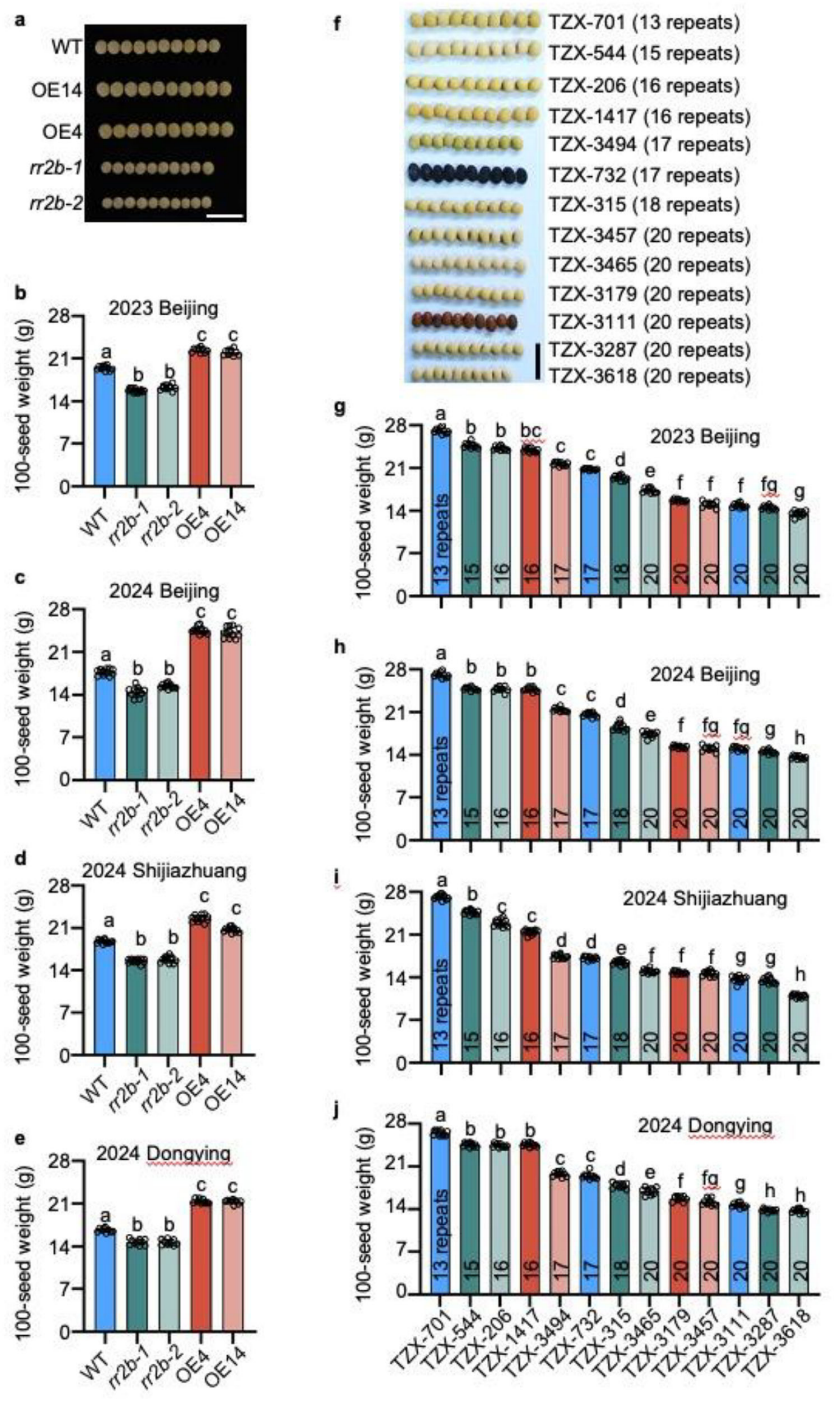
*RR2b* positively regulates soybean seed size. (a) Seed size for W82, *RR2b* OE, and *rr2b* knockout lines. Scale bars = 2 cm. (b–e) Quantification of 100‐seed weight for W82, *rr2b* knockout and *RR2b* OE lines grown at multiple field sites in 2023 and 2024. (f) Seed size of germplasm containing various insertion copies in the *RR2b* promoter. The numbers of insertion copies are listed in brackets. (g–j) Quantification of 100‐seed weight for germplasm containing various insertion copies in the *RR2b* promoter grown at multiple field locations in 2023 and 2024. Data are presented as means ± SD, and different letters indicate statistically significant differences at *p* < 0.05 by one‐way ANOVA analysis with Tukey's test. For (g–j), the numbers on the bars refer to the copy numbers of the (ATT) insertion.

### 2.5 The ABIG1–RR2b–bHLH63 Pathway Regulates Soybean Nodulation

As a previous study showed that RR11d, another type‐B response regulator in soybean, negatively regulates nodulation [22], we then investigated the roles of RR2b in soybean nodulation. Both *rr2b* mutant alleles have more root nodules, and *RR2b* OE plants have fewer nodules compared to WT controls, all with comparable root architecture (Figure S10a,b). These results suggested that RR2b is a negative regulator of soybean nodulation. Further study showed that several nodulation marker genes, including *NINa*, *NINb*, *ENOD40‐1*, *NSP1a*, and *NSP2a*, were up‐regulated in *rr2b* mutants and down‐regulated in *RR2b* OE plants relative to WT (Figure S11a–e). In CUT & Tag datasets, we also identified certain known nodulation‐pathway genes, including *CEP9* (*C‐TERMINALLY ENCODED PEPTIDE 9*) [36] and *NRLK* (*LEUCINE‐RICH REPEAT PROTEIN KINASE FAMILY PROTEIN*) [37] (Figure S11f,g). EMSA confirmed that RR2b–GST could bind in vitro to *cis*‐elements in the promoters of *CEP9* and *NRLK* (Figure S11h,i) and RR2b could down‐regulate *CEP9* and *NRLK* promoter activity in dual‐reporter assays (Figure S11j,k). Consistent with this, *CEP9* and *NRLK* expression is higher in *rr2b* mutants and lower in *RR2b* OE plants compared to WT (Figure S11l,m). These results support that RR2b represses soybean nodulation via these genes.

To identify the interaction protein partners of RR2b, a yeast two–hybrid assay was performed, and 50 candidates were identified (Table S4). Among them, a nuclear–localized basic helix‐loop‐helix transcription factor, bHLH63, garnered our interest. The direct interaction between RR2b and bHLH63b was confirmed in yeast (Figure S12a), and this was validated using bi‐molecular fluorescence complementation (Figure S12b), luciferase‐complementation imaging in tobacco leaves (Figure S12c), and co‐immunoprecipitation experiments in soybean hairy roots (Figure S12d). Toward understanding the functional relevance of the RR2b–bHLH63b interaction, *bHLH63b* was over‐expressed in transgenic hairy roots, resulting in fewer nodules (Figure S12e,f). Dual‐luciferase reporter assays demonstrated that bHLH63b could down‐regulated *NINa* promoter activity, similar to the effect observed with RR2b (Figure S12 g,h). These data indicate that RR2b could inhibits soybean nodulation along with its interaction protein partner bHLH63b.

Finally, we performed yeast one‐hybrid screening to identify the upstream regulatory *trans*‐element(s) of the *RR2b* promoter. Among the 26 candidates, one of the most frequently occurring candidate genes, ABIG1 (ABA INSENSITIVE GROWTH 1), a homolog of the abiotic‐stress‐related transcription factor ABIG1 in Arabidopsis [38], captured our attention (Table S5). ABIG1 could indeed activate the *RR2b* promoter in yeast (Figure S12i), and chromatin immunoprecipitation (ChIP)‐qPCR confirmed that ABIG1–Myc can bind to the *RR2b* promoter in soybean hairy roots (Figure S12j). Dual‐luciferase reporter assays showed that ABIG1 repressed the *RR2b* promoter (Figure S12k) and *ABIG1* over‐expression in transgenic hairy roots results in more nodules (Figure S12l,m), like *rr2b* knockout mutants (Figure S10a,b), indicating that ABIG1 positively regulates soybean nodulation by repressing *RR2b*. In sum, our data suggested that a pathway involving *ABIG1*, *RR2b*, and *bHLH63* acts to regulate soybean nodulation.

## 3 Discussion

In this study, we demonstrated that RR2b positively affects soybean grain yield by increasing seed size, while negatively regulating blight resistance by suppressing the ROS burst typical of PTI, and negatively affecting nodulation, a special type of plant‐microbe interaction. Although symbiotic nitrogen fixation can provide the nitrogen for legumes, there is also a trade‐off between nodule formation and yield [39]. Excessive nodulation can impair plant growth and yield by consuming too much photosynthates [40]. Therefore, the molecular mechanism by which RR2b functions in the complicated trade‐off among nitrogen fixation, yield, and disease resistance remains to be further elucidated in future studies. This transcription factor was artificially selected during soybean domestication based on its transcript level and balances yield and blight resistance, making it a promising target for optimizing both soybean yield and resistance (Figure S13). Given the importance of plant hormones in agricultural production, we also analyzed the selection patterns of genes involved in other classical plant hormone pathways, including all genes in the auxin, ethylene, ABA, and GA pathways. Interestingly, we found that only nine ABA pathway‐related genes were simultaneously selected during both domestication and improvement, and eight of them were located at the same locus as *RR2b* (Table S6). This suggests that multiple genes within the selective sweep regions containing *RR2b* might have been co‐selected, playing important roles in soybean domestication and improvement. Further mechanistic elucidation of other genes in this region will enhance our understanding of the selective sweep.

In Arabidopsis, type‐B ARRs exhibit significant functional redundancy, whereby single or double mutants typically do not display obvious developmental abnormalities [41, 42]. Therefore, the phenotypic anomalies in plant height, nodulation, yield, and blight resistance observed in the *rr2b* single mutant suggest that this gene plays a particularly important role in soybean growth, development, and resistance. This functional differentiation and specificity are also reflected in the relatively independent phylogenetic position of RR2s within the entire soybean type‐B RR family (Figure S4). In this study, we identified that the DNA motif bound by RR2b is TTGGAAT, which is different from the GGATT motif previously identified as the binding site for type‐B RRs in Arabidopsis and other leguminous plants [22, 26, 28]. This suggests that RR2b may not only function through the typical activation of cytokinin signaling pathways but could also bind to the promoters of other downstream genes to regulate additional biological processes. Consistent with this, *RR2b* is widely expressed in multiple soybean tissues, and its protein products can physically interact with various interacting partners, as well as the promoters of downstream genes (File S1). These context‐dependent changes in its interacting partners explain why *RR2b* can exert this dual function as both an activator and a repressor. For example, changes in cell‐wall‐synthesis‐related genes and *KNOX* genes in the *rr2b* mutant may contribute to increased plant height, which is a typical cytokinin‐activator phenotype. In contrast, the enhanced ROS burst and improved disease resistance following *RR2b* mutation indicate that it functions as a repressor in soybean disease‐resistance processes. The N‐terminal domain of type‐B ARRs, which serves as the signal‐reception region, can inhibit the transcriptional activity of the C‐terminal domain, which acts as the transcriptional activation domain [27, 28]. In this study, we identified a very short 26‐amino‐acid domain in the C‐terminal that is sufficient and necessary for the transcriptional activity of RR2b, providing new insights for further elucidating the mechanism of RR2b's function.

Artificial selection of crops occurs in two stages, domestication and improvement. Early domestication steps change fundamental characteristics of the wild progenitor, resulting in landrace varieties, which provide the genetic materials for breeders to later select improved varieties and inbred lines. Subsequent diversification or improvement emerges as crops spread to different regions and undergo more conscious selection that can enhance traits which influence agricultural productivity and performance, including yield and resistance to stress [43]. In response to changing environments during the early domestication steps, breeders have selected plants that are better adapted to local conditions. This selection process has led to the emergence of landraces, which are traditional varieties chosen by farmers for their suitability to local conditions and food preferences. Although these varieties typically exhibit low yield potential, they are often characterized by genetic diversity and resilience to environmental stresses [44]. Here, a particularly intriguing phenomenon is that the high‐yield *RR2b* haplotype HT2 was initially lost during soybean domestication from the wild relative to landrace varieties, followed by a decline in the prevalence of the highly blight‐resistant haplotype HT3 during improvement from landraces to cultivar varieties. One possible explanation is that, in the early stages of cultivation, farmers had limited effective methods for disease control. Therefore, it is essential to first preserve varieties that are disease‐resistant, followed by the selection of varieties that achieve an optimal balance between disease resistance and yield.

To date, research on the selection of disease‐resistant traits during crop domestication remains fairly limited. Zhou et al. [14] demonstrated that the region harboring the root‐knot nematode resistance locus *Rhg1* in soybean was subject to selection. Additionally, a pan‐transcriptome analysis revealed that disease‐resistance genes were also selected during barley domestication [45]. The prevailing view suggests that the reduction of genetic diversity during domestication may lead to a ‘broad susceptibility’ to newly emerging herbivores and pathogen strains, thereby compromising long‐term crop sustainability [46, 47, 48]. In legumes, half of the annotated resistance‐related sequences in soybean and chickpea were lost by domestication and subsequent improvement [14, 49]. Therefore, retrieving ancestral disease‐resistance genes lost due to genetic bottlenecks during artificial selection from wild relatives or landraces, or developing disease‐resistance genes preserved in cultivated varieties, such as *RR2b* identified here, presents a feasible strategy for enhancing soybean disease‐resistance breeding.

Both plants and animals produce ROS, which not only have a direct antimicrobial effect but also serve as signaling molecules to activate pathogen‐resistance responses [50]. Exogenous application of a high concentration of cytokinins or increasing the content and signaling of endogenous cytokinins through genetic means in Arabidopsis can enhance defenses against pathogens. In this process, ARR2 plays positive roles by regulating the salicylic acid pathway and ROS homeostasis, which involves apoplastic peroxidases PRX4, PRX33, PRX34, and PRX71, but not the RBOH class of NADPH oxidases [32, 33]. However, our results suggest a negative role for RR2b in soybean resistance through an RBOH‐dependent ROS burst (Figure 4). This discrepancy might be caused by the different mechanisms of ROS generation. Indeed, RR2b plays distinct roles in various physiological processes and signaling pathways. For instance, here, RR2b activates the cytokinin pathway in soybeans, akin to its homolog in Arabidopsis [26]. Meanwhile, it also functions as a transcriptional repressor, inhibiting disease‐resistance pathways and root‐nodule formation in soybean. Likewise, mutation of a B‐type RR, *MtRRB3*, lead to fewer root nodules in *M. truncatula*, indicating its role as a positive regulator of the nodulation pathway [51]. Disruption of *RRB12* in *L. japonicus* caused a reduction in nodulation [52]. In contrast, our results indicate that RR2b represses the nodulation pathway, despite its capacity to activate cytokinin signaling as a transcription factor, which aligns with a previous study in soybean [22]. This inconsistency may be attributed to species differences or variation between determinate and indeterminate root nodules.

Crop domestication is typically examined from evolutionary and genetic perspectives, primarily focusing on key agronomic traits referred to as domestication‐related traits. However, there has been only limited research on ecological domestication traits, such as growth and resource‐acquisition rates, interactions with microbes, insects, and pollinators. The significance of such traits lies not only in yield (i.e., plant fitness) but also includes various ecological effects, such as carbon and nitrogen sequestration, nutrient use and cycling in the soil, the ecological balance amongst other community members, as well as implications for climate change. Relatively fewer ecological domestication trait studies have focused on crop adaptation to climate change and photoperiod [53, 54, 55, 56]. Here, we revealed that the artificially selected transcription‐factor gene *RR2b* in the cytokinin pathway orchestrates domestication‐related traits (i.e., yield) and ecological domestication traits (i.e., pathogen resistance) during soybean domestication. When pathogens invade agricultural systems and lead to disease, the balance between yield and disease resistance becomes important, and rapid and precise regulation at the level of transcription may represent a shortcut to achieve this balance.

The growth–defense trade‐off is regarded as one of the fundamental principles of ‘plant economics’ [57]. Strong trade‐offs caused by gene pleiotropy are one of the biggest barriers to crop improvement. For example, the *gl4* mutation in the domestication gene in African cultivated rice *O. glaberrima* reduces seed shattering, albeit at the expense of seed size [58]. In Asian rice *O. sativa*, *semi‐dwarf1* (*sd1*) reduces lodging risk but also decreases plant biomass and nitrogen‐use efficiency [59]. A gain‐of‐function of *IPA1* (*IDEAL PLANT ARCHITECTURE 1*) results in larger panicles in rice and enhanced disease resistance, but leads to fewer tillers [60]. In this study, pleiotropy of *RR2b* results in a trade‐off between yield and blight resistance. There are three feasible approaches to decouple this pleiotropy. One is to target a regulatory element to modify its *cis*‐regulatory regions, thereby separating the multiple functions of RR2b by altering the developmental stages and tissue‐specificity of its expression, as performed in other crop [61]. The second entails identifying distinct protein‐function domains that may contribute to pleiotropy and constructing various truncated or mutated versions of this transcription factor to fulfill the necessary functions under different environmental conditions. Our identification of the minimal, sufficient, and necessary sequence for RR2b transcriptional‐regulation activity provides the possibility for this approach (Figure 2g). The third approach, identifying and characterizing more downstream target genes of RR2b, clarifying their relevant traits, and analyzing the mechanisms acting in different processes, will also provide valuable new clues for breaking the linkage drag of multiple traits. In future soybean breeding, disrupting *RR2b* pleiotropy and addressing linkage drag will offer new strategies to decouple the yield‐disease resistance tradeoff, ultimately leading to the development of high‐yielding and disease‐resistant varieties.

## 4 Materials and Methods

### 4.1 Plant Materials and Growth Conditions

*Glycine max* cv. Williams 82 (W82) was used as the wild type for gene cloning, gene expression, and hairy‐root transformation experiments. Soybean seeds were sown in sterile vermiculite for germination. The plants were propagated in a greenhouse (16‐h light/8‐h dark cycle at 25°C and 65% relative humidity) equipped with LED lights at 160 µmol m^−2^s^−1^ light intensity. After germination for 5 d, seedlings were inoculated with a suspension of *Bradyrhizobium japonicum* USDA110 (OD_600 nm_ = 0.08) and root nodules were examined 21 d later.

### 4.2 Soybean Genomic DNA Extraction

DNA was extracted from the leaves using a CTAB (cetyltrimethylammonium bromide)‐based method. Leaves were ground with 600 µL of CTAB solution and incubated in an oven at 65°C for 20 min. Three hundred microliters of chloroform were added, and the mixture was shaken vigorously before centrifugation at 13 000 × g for 10 min. Subsequently, 350 µL of the supernatant was aspirated, and 700 µL of anhydrous ethanol was added, mixed well, and then transferred to –20°C for 30 min. A 13 000 × g centrifugation step was then performed for 10 min. The supernatant was discarded, and the sediment was washed in 70% v/v ethanol twice. The samples were then centrifuged at 13 000 × g for 5 min, and the supernatant was discarded. Each sample was dissolved in 100 µL of water after desiccation.

### 4.3 RNA Extraction and Quantitative PCR Analysis

Total plant RNAs were extracted using the TRIzol Up Kit (TransGen, Beijing, China). Genomic DNAs were removed, and cDNA was synthesized from 500 ng of RNA using the HiScript III RT SuperMix for qPCR with gDNA wiper (Vazyme, Nanjing, China). The RT–qPCR reaction was performed using Taq Pro Universal SYBR qPCR Master Mix (Vazyme, Nanjing, China), and the primer pairs used for RT–qPCR are listed in Table S6. The PCR reactions contained 5 µL of 2 × Taq Pro Universal SYBR qPCR Master Mix, 0.2 µL each of forward and reverse primer (10 µm), 1 µL of template cDNA (50 ng/µL), and 3.6 µL of RNase‐free ddH_2_O. The PCR protocol consisted of an initial denaturation at 95°C for 1 min (1 cycle), followed by 40 cycles of denaturation at 95°C for 5 s and annealing/extension at 60°C for 30 s, with a final melting curve analysis. *GmELF1b* (*Glyma.02G276600*) was used as the reference gene for normalization of relative expression [62]. Fold change was calculated from the 2^−ΔΔCt^ values.

### 4.4 Plasmid Construction

Construction of *GUS* reporter plasmids was done using genomic DNA obtained above as a template, and PCR reactions were performed using primer pairs to obtain a series of promoter sequences (∼3000 bp) carrying different *RR2b* haplotypes. The promoter sequences were cloned into the intermediate vector pGWCm through the In‐Fusion reaction system (Vazyme, Nanjing, China). Using Gateway LR cloning, the intermediate vector was subjected to an LR reaction with the vector pBGWFS7 to obtain *GUS* reporter vectors.

Construction of *LUC* reporter plasmids using genomic DNA as a template, PCR reactions were performed using primer pairs to obtain a series of promoter sequences (∼3000 bp). The promoter sequences were cloned into the CP461 vector through the In‐Fusion reaction system (Vazyme, Nanjing, China).

W82 cDNA was used as a template for PCR using primer pairs to obtain the target coding sequences. The above coding sequences were cloned into pTF101 through the In‐Fusion reaction system (Vazyme, Nanjing, China) to obtain an over‐expression vector in which RR2b was fused to a triple FLAG tag (3xFLAG) at the C‐terminus.

### 4.5 Genetic‐Diversity Analysis

Published SNPs and InDels data were used for diversity analysis of *RR2b* [14]. The SNPs and InDels with missing data >10% or MAF<5% were filtered. Accessions were divided into three populations: *G. soja*, landraces, and cultivars. Nucleotide diversity (π) was calculated by 20‐Kb window with 2‐Kb sliding step on the genome. After filtering the windows with <10 SNPs/InDels in wild and 0 SNPs/InDels in cultivated populations, we calculated the ratio of diversity (π_wild_/π_cultivated_) for each window. *F*_ST_ values were calculated by a 20‐Kb window with a 2‐Kb sliding step using VCF tools [63], to estimate the pairwise genomic differentiation between wild and cultivated populations of soybean. The top 5% values of the ratio of diversity and *F*_ST_ were used to identify selective sweeps.

### 4.6 Haplotype Analysis of RR2b

SNPs and InDels analyses of the 3‐kb promoter region and full‐length coding sequences of *RR2b* were done using 296 sequenced varieties in a natural population [64]. SNPs and the InDels were filtered by applying a MAF >5% cutoff, missing rate < 10%, synonymous SNV, and non‐functional SNP mutation [65]. The hundred seed weight and seed weight per plant of *G. max* accessions of different *RR2b* haplotypes were estimated to clarify the effect of *RR2b* during domestication. Data were collected from the SoyOmics database (https://ngdc.cncb.ac.cn/soyomics). Significance test was done using Student's *t*‐test and Wilcoxon rank‐sum test.

### 4.7 Hairy‐Root Transformation and Generation of Stable Over‐Expression and Gene‐Edited Soybean Lines

The over‐expression plasmids were transformed into *Agrobacterium rhizogenes* K599, which was used to infect soybean seedlings to generate transgenic hairy roots following a previously published protocol [66].

For CRISPR–Cas9 vector construction, the pYLCRISPR/Cas9‐DB vector was used as previously reported [67] and small guide RNAs were designed using CRISPR‐P2.0 (http://crispr.hzau.edu.cn/CRISPR2/) [68]. Two highly specific sequences in the exonic region of *RR2b*, TCGAGTTCTCCTCTGAAAGCCGG and CTTGCCTTATGATCCTTGAGAGG, were selected as potential targets for gene editing. The *RR2b* knock‐out vector was transformed into *A. tumefaciens* EHA105. *A. tumefaciens*‐mediated genetic transformation was used to obtain T_1_‐generation *RR2b* knockout lines. In order to analyze whether *RR2b* was edited and its editing effect, its integrity in the T_1_ generation was detected by DNA sequencing. T_2_ and T_3_ Cas9‐free homozygotes were analyzed for phenotypes, cytokinin responses, and agronomic traits.

For generating over‐expression stable transgenic plants, W82 cDNA was used as a template for PCR to obtain the *RR2b* coding sequence for cloning into the pWMV078 vector through the In‐Fusion reaction system. The vector was transformed into *A. tumefaciens* EHA105 and genetic transformation of soybean was performed based on previously protocols [69, 70]. Putative transgenic plants were genotyped by PCR, and lines with at least 5‐fold higher expression than that of the wild type were deemed to be over‐expression plants.

### 4.8 Analysis of RR2b Promoter Haplotype Transcriptional Activity

PCR was performed to obtain a series of promoter sequences (∼3000 bp) carrying different *RR2b* haplotypes. The promoter sequences were cloned into the intermediate vector pGWCm through the In‐Fusion reaction system (Vazyme, Nanjing, China). Using Gateway LR cloning, the intermediate vector was subjected to an LR reaction with the vector pBGWFS7 to obtain *GUS* reporter vectors. The above vectors were transformed into *A. tumefaciens* GV3101, mixed in equal proportions with a strain harboring the P19 suppressor of silencing, and uniformly infiltrated into the lower epidermis of *Nicotiana benthamiana* leaves, and maintained for 48 h to obtain transiently transgenic leaves. GUS expression was analyzed by histological staining [69].

To verify the transcriptional activity of the different haplotypes, genomic DNA was used as a template for PCR amplification of promoters carrying the different *RR2b* haplotypes. The above sequences were cloned into the CP461 vector through the In‐Fusion reaction system (Vazyme, Nanjing, China), obtaining the dual‐luciferase reporter plasmid *RR2b^HT^_pro_:LUC*. The above vectors were transformed into *A. tumefaciens* GV3101, mixed with P19 in equal proportions, and uniformly infiltrated into the lower epidermis of *N. benthamiana* leaves, and maintained for 48 h to obtain transiently transgenic leaves. Dual luciferase assay reagent (Beyotime, Shanghai, China) was used for luciferase imaging using the sea kidney luciferase gene (*RENILLA*) as an internal control.

### 4.9 Yeast Two‐Hybrid (Y2H) Assay

For testing auto‐activation in yeast, the full‐length coding sequence of *RR2b* and different‐length coding sequences were obtained by PCR. By homologous recombination, PCR products were ligated into pGBKTT7 vector, and different lengths of BD vector and AD vector were co‐transformed into the Y2H Gold strain and grown on SD/–Trp/–Leu (DDO) medium. Interactions were assayed by spreading 3 µL of suspended transformed yeast on plates containing SD/–Trp/–Leu/–Ade/–His (QDO) medium. The interactions were observed after 3–4 d of incubation at 30°C.

To identify RR2b‐interacting proteins, we performed a Y2H screening using the GAL4 yeast two–hybrid system (Coolaber, Beijing, China). RNA was extracted from all tissues of W82 and used to make cDNA for the Y2H library, which was constructed by OE Biotech (Shanghai, China). The Y2H strain containing the BD vector with the full–length coding sequence of *RR2b* was grown on SD/–Trp/–Leu (DDO) medium in a low‐rate co‐culture with the AD library strain. Clones on DDO were spread with 3 µL of suspended transformed yeast on plates containing SD/–Trp/–Leu/–Ade/–His (QDO) medium. Clones on QDO were sequenced and compared on SoyBase to obtain the candidate interacting proteins.

The full‐length coding sequence of *RR2b* was amplified by PCR, and cloned into the pGBKT7 (bait) vector to generate the BD–RR2b plasmid. The full‐length coding sequence of *bHLH63b* was amplified and inserted into the pGADT7 (prey) vector, yielding plasmid AD–bHLH63b. Bait plasmids were linearized and transformed into Y2H Gold. Positive cells were then transformed with the AD–bHLH63b plasmid. Transformation was confirmed by growth on SD/–Trp/–Leu (DDO) medium. Interactions were assayed by spreading 3 µL of suspended transformed yeast on plates containing SD/–Trp/–Leu/–Ade/–His (QDO) medium. The interactions were observed after 3–4 d of incubation at 30°C. Primers used are listed in Table S8.

### 4.10 Bimolecular Fluorescence‐Complementation (BiFC)

The coding sequences of the target proteins were obtained using a cDNA template and cloned into the pCambia2300‐NYFP and pCambia2300‐CYFP vectors through the In‐Fusion reaction system. The vectors were transformed into *A. tumefaciens* GV3101, mixed with P19 in equal proportions, and uniformly infiltrated into the lower epidermis of *N. benthamiana* leaves, and incubated for 48 h to obtain transgenic leaves. Leaves were imaged using a ZEISS LSM 980 super‐resolution confocal microscope [65].

### 4.11 Luciferase Complementation Imaging (LCI)

*RR2b* was cloned in‐frame and upstream of the sequence encoding the N‐terminal half of *LUC* in pCAMBIA1300‐NLUC, while *bHLH63b* was cloned in‐frame and downstream of the C‐terminal half of *LUC* in pCAMBIA1300‐CLUC. The resulting vectors, as well as empty plasmids, were introduced into *A. tumefaciens* GV3101, and transiently expressed in *N. benthamiana* leaves as described above. Luciferase activity from *N. benthamiana* leaves was detected 2 d after infiltration with a chemiluminescence imager with a cooled CCD camera.

### 4.12 Co‐IP Assay

The RR2b–GFP vector and bHLH63b–3xFLAG vector were co‐expressed in *N. benthamiana* leaves, and total protein were extracted in buffer (50 mm HEPEs, pH 7.5, 150 mm NaCl, 10 mm EDTA pH 8.0, 1% v/v Trion X‐100, 10% w/v sucrose, 2 mm DTT, 1 mm PMSF, 25 µm MG132, 1× protease inhibitor cocktail) and incubated with 20 µL anti‐GFP beads (AlpaLifeBio, Guangdong, China) for 2 h at 4°C. Beads were washed with buffer three times, to which 50 µL of 1× SDS–PAGE sampling loading buffer was added and heated at 95°C for 10 min. Proteins co‐precipitated and extracted from plant tissues were separated by 10% SDS–PAGE and transferred to PVDF membranes. After blocking with 5% non‐fat milk in TBST for 1 h at room temperature, membranes were incubated overnight at 4°C with primary antibodies (diluted 1:3,000 in blocking buffer), followed by HRP‐conjugated secondary antibodies (1:10 000 dilution, 1 h at room temperature). Signals were detected using an ECL substrate and visualized with a chemiluminescence imaging system [71].

### 4.13 Yeast One‐Hybrid (Y1H) Assays

A 500‐bp fragment of the *RR2b* promoter was amplified by PCR, and cloned into the pAbAi (bait) vector to generate a pAbAi‐*_pro_RR2b* plasmid. The full‐length *ABIG1* coding sequence was amplified and inserted into the pGADT7 (prey) vector, yielding plasmid AD–ABIG1. The bait plasmids were linearized and transformed into the yeast strain Y1H Gold. Positive cells were then transformed with the AD–ABIG1 plasmid. The DNA–protein interaction was determined based on the growth ability of the co‐transformants on SD/–Leu medium with 200 ng/mL Aureobasidin A (AbA) (CAS 127785‐64‐2, Yeasen).

### 4.14 CUT & Tag‐seq

CUT&Tag was performed using Hieff NGS G‐Type In‐Situ DNA Binding Profiling Library Prep Kit for Illumina (Yeasen, Shanghai, China) according to the manufacturer's protocol and was performed as described previously [72]. Wild‐type W82 and *RR2b*–*3xFLAG* OE plants were grown in vermiculite for 5 d and then inoculated with *B. japonicum* USDA110 (OD_600 nm_ = 0.08), and fresh root tissue was collected after 3 d of continued growth. The nuclei of soybean root tissues were extracted and incubated with primary and secondary antibody at room temperature for 2 h and 0.5–1 h, respectively. The samples were incubated at 37°C for 1 h for transposase activation, further digested with proteinase K, and DNA was recovered by magnetic beads. The DNA library was amplified and purified, and sent to NovaSeq for sequencing at Novogene (Beijing, China).

### 4.15 ChIP‐qPCR

ChIP‐qPCR was performed as previously described [66]. In brief, K599 strain carrying *UBI_pro_::ABIG1–Myc* was injected to hypocotyl of 5‐day‐old W82. Hairy roots were collected at 19 dpi (day‐post‐injection) and cross‐linked with 1% formaldehyde for 20 min under vacuum, followed by neutralization using 0.125 m glycine. Then, those hairy roots were ground into powder, and the nuclei were isolated via sucrose density gradient centrifugation. After that, the samples were sonicated on ice using a sonicator and centrifuged. One‐tenth volume of supernatant was reserved as input, and the remains was performed immunoprecipitation using Myc–magnetic beads (Lablead, Beijing, China) or ChIP–grade protein A+G magnetic beads (control) (Beyotime, Shanghai, China). After a thoroughly wash of the beads, DNA was extracted by 5% Chelex–100 (Biorad, California, USA) and phenol‐chloroform‐isoamyl alcohol mixture (Acmec, Shanghai, China) for qPCR analysis. *GmABCT* (*Glyma.12G020500*) was used as an internal control. Fold enrichment was calculated by normalizing the specific *proRR2b* region to *GmABCT*, relative to the Protein A+G control. The specific primers used in this experiment are listed in Table S8.

### 4.16 RNA‐seq Sample Preparation and Sequencing

Roots of WT, *rr2b* knockout, and *RR2b–3xFLAG* OE plants were collected for RNA‐seq analysis. Three biological replicates were performed for each genotype. The Illumina HiSeq 2000 platform was used to generate 150‐bp paired‐end reads. Detailed bioinformatic analyses were performed as previously described [65]. Briefly, the high‐quality sequencing reads were mapped to the reference genome with Hisat (v.2.2.1), and gene‐expression counts were calculated using StringTie (v.1.3.4d). Differential gene‐expression analysis was done using R–edgeR library (https://bioconductor.org/packages/release/bioc/html/edgeR.html).

### 4.17 Transient Dual‐Luciferase Assay

To validate RR2b target genes, promoter sequences of soybean *NINa*, *CEP9*, *NRLK, PPR40*, *RbohA*, *RbohC*, *RbohD, RR5a, RR9c*, *ATKH8*, and *LRR4* genes were obtained by PCR using W82 genomic DNA. PCR products were cloned into the CP461 vector through the In‐Fusion reaction system (Vazyme, Nanjing, China) to obtain dual‐luciferase reporter vectors. pTF101 was used to construct the over‐expression vector *35S_pro_:RR2b* with the full‐length coding sequence of *RR2b*. The above vector was transferred into *A. tumefaciens* GV3101, mixed with P19 in equal proportions, and uniformly infiltrated into the lower epidermis of *N. benthamiana* leaves, and maintained for 48 h to obtain transiently transgenic leaves. Dual‐luciferase assay reagent (Beyotime, Shanghai, China) was used for luciferase imaging using the *RENILLA* gene as an internal control.

### 4.18 Electrophoretic Mobility Shift Assay (EMSA)

EMSA was performed using the LightShift Chemiluminescent EMSA Kit (Beyotime, Shanghai, China) according to the manufacturer's protocol and as described previously [73]. GST and GST–RR2b were expressed in *E. coli* Rosetta and purified. The binding activity of the protein was analyzed using an oligonucleotide probe labeled with biotin at the 5′ end (Sangon Biotech, Shanghai, China). For unlabeled probe competition, 20 and 200‐fold molar excesses of unlabeled probes were added to the reactions.

### 4.19 Reactive‐Oxygen Burst Measurements

ROS production assay was performed as previously described [74]. Briefly, wild‐type W82, *rr2b* knockout and *RR2b‐3xFLAG* OE plants were grown in vermiculite for 4 d and fresh root tissue was cut into pieces about 3 mm long and transferred to a 96‐well plate with 200 µL of ddH_2_O. Incubation in low light overnight allowed reactive oxygen species produced by tissue damage to quench themselves. The next day, water was discarded, and 200 µL of a buffer solution containing 20 µm luminol and 10 µg/mL peroxidase with the appropriate concentration of elicitors such as 100 nm flg22 was added. Detection of fluorescent signals was performed using an EnVision plate reader (Perkin Elmer).

### 4.20 Disease‐Resistance Testing

Disease‐resistance testing was performed as previously described [74]. Briefly, soybean plants were grown in vermiculite for 14 d, and the first ternately compound leaves were selected for inoculation. Inoculation with *Pseudomonas syringae* pv. *glycinea* (OD_600 nm_ = 0.05) was performed on both leaf surfaces and incubated at high humidity for 2 d. Bacteria in leaves were extracted with ddH_2_O, and 20 µL of the suspension was spread on TSA medium, then the number of colonies were counted 2 d later after growth at 28°C.

### 4.21 Trait Measurement and Plot Field Tests

W82, *rr2b* knockout and *RR2b–3xFLAG* OE plants were grown in fields in Beijing, Shijiazhuang and Dongying (China) under natural conditions in 2023 and 2024. Soybean germplasms were also grown in the same fields. Each plot had three replicates of the same size, six rows were planted in each plot, and plants were collected from the middle four rows for yield testing. The length of each plot was 3 m (Beijing) or 5 m (Shijiazhuang and Dongying), and the spacing between rows was 50 cm. Three weeks after sowing, seedlings were manually thinned to achieve a plant density of 1350 plants per 100 m^2^. Plant height was measured at the maturity stage. Fully filled grains were used for measuring 100‐seed weight.

### 4.22 Phylogenetic Analysis

The protein sequences of *A. thaliana* were obtained from The Arabidopsis Information Resource (TAIR) database (https://www.arabidopsis.org/). The protein sequences of *G. max*, *L. japonicus*, and *M. truncatula* were obtained from the Phytozome database (https://phytozome‐next.jgi.doe.gov/). The phylogenetic tree was built with MEGA12, using the neighbor‐joining method with the bootstrapping value set at 1000 replications.

### 4.23 Statistical Analysis

Histograms were made using GraphPad Prism 8.2.1, data are plotted as means ± SD as indicated, and every dot represents each sample. A one‐way analysis of variance (ANOVA) was conducted, followed by the Tukey multiple‐comparisons test to compare the means of each column with the means of all other columns. Statistical significance between a treatment with a control was analyzed by two‐tailed, unpaired Student's *t*‐test. Categorical data associations were evaluated using Fisher's exact test, with exact *P* values calculated based on hypergeometric distributions.

### 4.24 Quantification of Endogenous CKs From Plant Tissues

Endogenous CKs were extracted and quantified as previously described [75]. Briefly, approximately 100 mg (fresh weight) plant tissues were homogenized in liquid nitrogen, weighed, and extracted with 90% methanol (MeOH) overnight at 4°C containing stable isotope labelled compounds as internal standards. The extracts were then centrifuged at 20 000 g for 15 min at 4°C, and the supernatant was collected. The crude extracts were loaded onto the connected MCX cartridges (Waters, USA), which had been activated using the method recommended in the product manual. The MCX cartridges were washed sequentially with 2% formic acid in 5% MeOH, 5% MeOH, and MeOH, followed by elution of CKs with 80% MeOH containing 5% NH_4_O. The eluate was dried under a nitrogen stream and then redissolved in 20% MeOH for UPLC‐MS/MS analysis. Analysis was performed on a UPLC instrument (Waters, USA) combined with a 6500 Qtrap MS system equipped with an electrospray ionization source (AB SCIEX, USA). The UPLC methods and MS parameters were set as reported previously [75].

## Author Contributions

Q.M., L.L., Z.T., and B.R. conceived the study, Q.M., Y.C., Y.G., X.H., Q.Y., Y.L., P.X., J.Y., and J.C. performed the experiments, Q.M., L.L., Z.T., and B.R. analyzed the data, B.R. wrote the manuscript with input from Q.M., L.L., and Z.T. All authors read the manuscript and agreed to its submission.

## Funding

This work was supported by the National Key Research and Development Program of China (2021YFF1000102) and the Chinese Academy of Sciences Project (XDB1580201).

## Conflicts of Interest

This work has been filed for patent applications with B.R., Q.M., Y.C., and X.H. listed as inventors. All other authors declare no competing interests.

## Supporting information




**Supporting File 1**: advs76615‐sup‐0001‐SuppMat.pdf.

**Supporting File 2**: advs76615‐sup‐0002‐Tables.xlsx.

## Data Availability

All data are available in the National Genomics Data Center (https://ngdc.cncb.ac.cn/), Beijing Institute of Genomics, Chinese Academy of Sciences, under the BioProject number PRJCA040019.
