# Supplementary material for: An Artificially Selected Cytokinin‐Pathway Transcription Factor Balances Soybean Yield and Pathogen Resistance
Source: Adv Sci (Weinh). 2026 Jul 20:e76615. Online ahead of print. doi: 10.1002/advs.76615 (PMC13383695; doi:10.1002/advs.76615)
Supplement: Supplementary file 1 — Supporting File 1: advs76615‐sup‐0001‐SuppMat.pdf. [file ADVS-9999-e76615-s002.pdf]

a

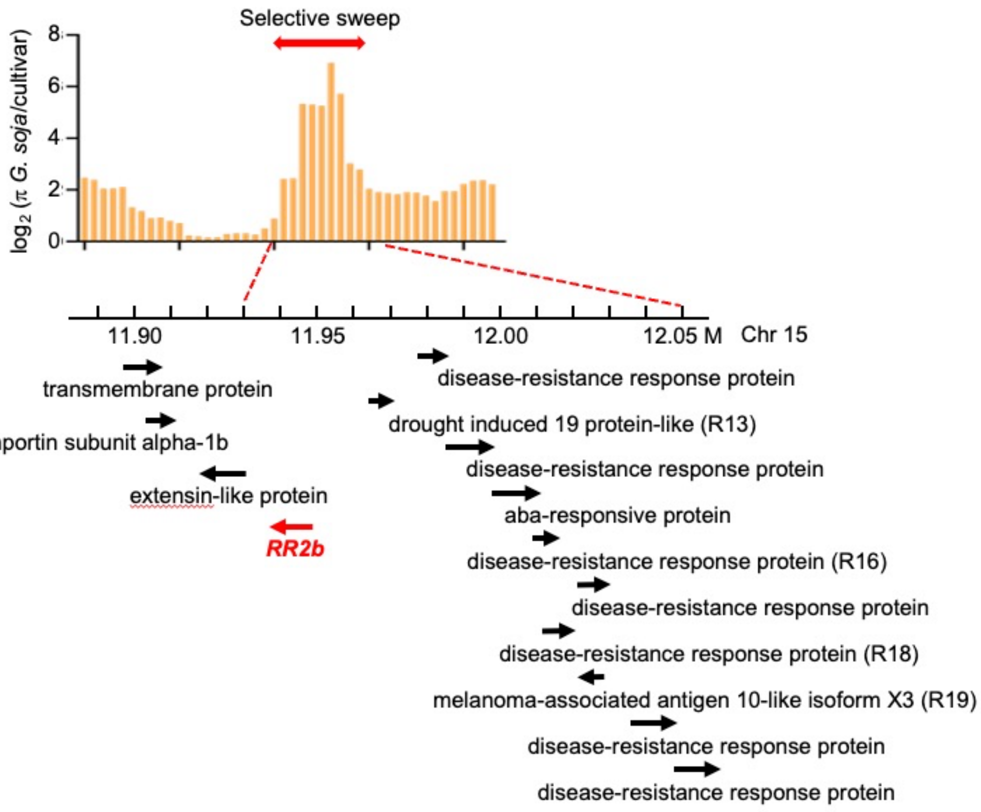

b

QTL Name	Chr. 15 (Mb)	Trait Name
Phytoph 5-2[76]	6.8–13.6	Reaction to *Phytophthora sojae* infection
SCN46-3[77]	11.6–13.7	Reaction to *Heterodera glycines*
Seed set 5-30[78]	6.8–16.6	Fertility related trait
SDS disease incidence 21-2[79]	9.4–16.6	Reaction to *Fusarium solani* f. sp. *glycines* infection
Asian Soybean Rust 2-4[80]	11.1–50.7	Reaction to *Phakopsora pachyrhizi* infection
Flower number 1-7[81]	11.1–50.7	Flower number trait
Seed genistein 11-3[82]	11.1–50.7	Seed genistein content
Seed glycitein 13-4[83]	11.1–50.7	Seed glycitein content
Seed isoflavone 11-10[83]	11.1–50.7	Seed isoflavone

Figure S1. Characterization of the *RR2b* locus in the soybean genome.

(a) *RR2b* is co-selected with clustered resistance genes during soybean domestication. Nucleotide diversity (π) at the *RR2b* locus is displayed with colored lines. Black arrows indicate the gene direction. (b) *RR2b* is situated in

several reported resistance/yield-related QTLs. References are superscript on the QTL names.

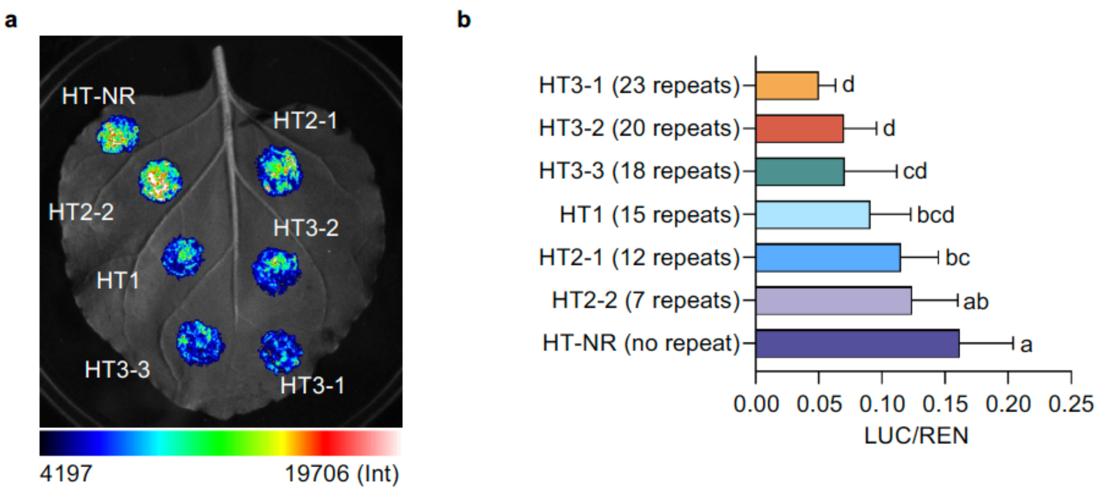

Figure S2. The number of insertions in the *RR2b* promoter dictates its activity.

(a) Chemiluminescent image of transient dual-luciferase assays of *RR2b* promoter activity from various haplotype groups. (b) Relative LUC/REN of promoters assayed in panel a. ‘HT-NR’ (‘no repeat’) is an artificial haplotype containing zero ATT insertion in the *RR2b* promoter. Data are means ± SD (n = 8). Three independent experiments were repeated with similar results. Different letters indicate statistically significant differences in a one-way ANOVA analysis with Tukey’s test ($p < 0.05$).

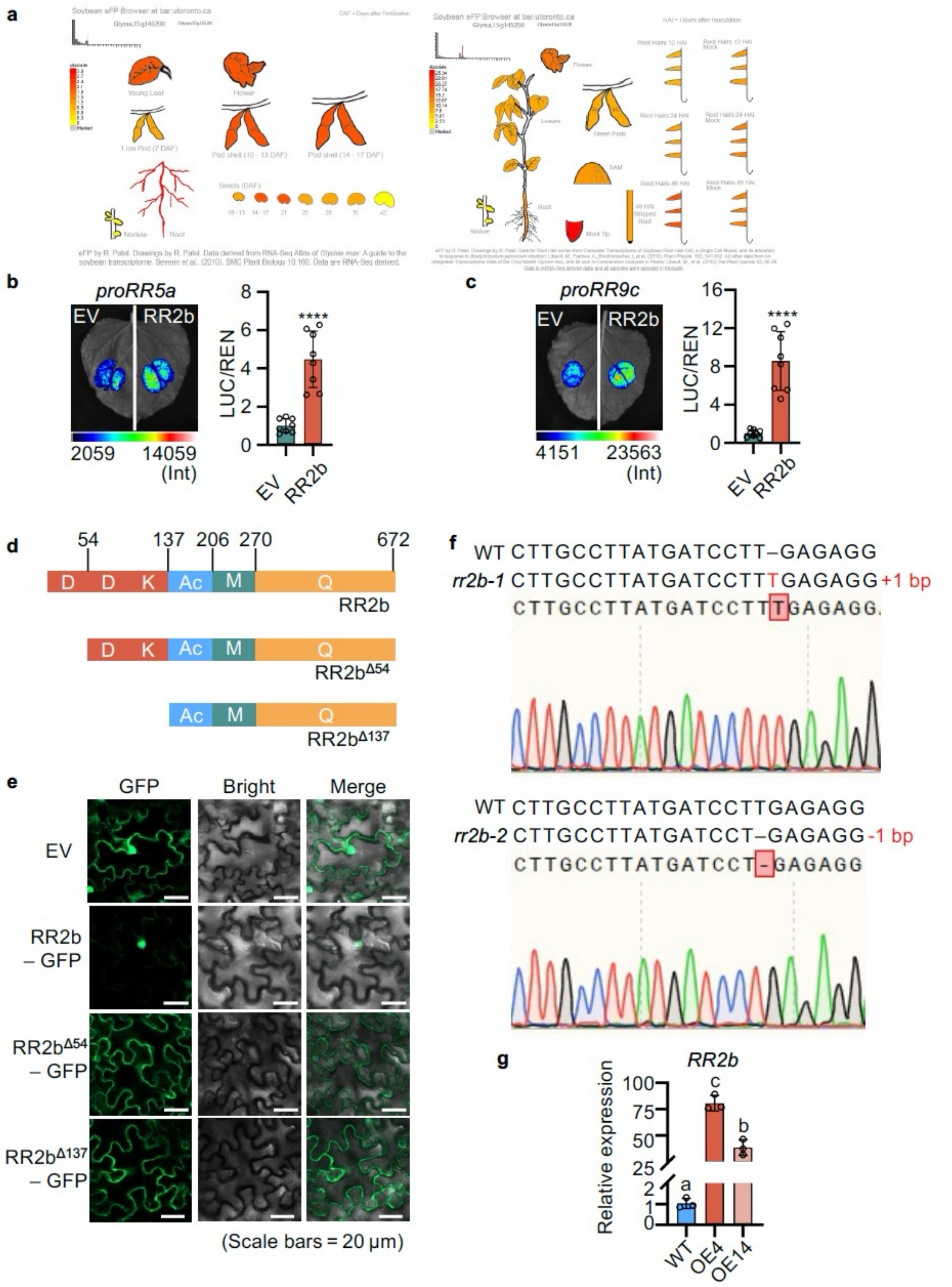

Figure S3. Characterization of *RR2b* tissue-level gene expression, RR2b domain structure and subcellular localization, and plant lines lacking or over-expressing *RR2b*.

(a) Expression pattern of *RR2b* in various tissues obtained from the Plant eFP

Viewer (https://bar.utoronto.ca/eplant_soybean/). (b, c) Transient dual-luciferase assay of RR2b activating cytokinin signaling pathway promoters of *RR5* (b) and *RR9c* (c). LUC/REN, ratio of firefly luciferase to *Renilla* luciferase activity. Data are means ± SD (n = 8). Three independent experiments were repeated with similar results. Asterisks indicate statistically significant differences relative to the EV control. Two–sided Student's *t*–test, ****$p < 0.0001$. (d) Schematic of RR2b domain organization. RR2b is the full-length version, RR2b$^{\Delta 54}$ is the first Asp-deletion version, and RR2b$^{\Delta 137}$ is the double Asp-deletion version. Ac, M and Q mark the acidic domain, the ARRM domain, and the glutamine-rich domain, respectively. (e) Subcellular localization of RR2b–GFP. Constructs ($35S_{pro}$*:GFP*, $35S_{pro}$*:RR2b–GFP*, $35S_{pro}$*:RR2b*$^{\Delta 54}$*–GFP* and $35S_{pro}$*:RR2b*$^{\Delta 137}$*–GFP*) were transformed into *N. benthamiana* leaf epidermal cells for confocal microscopy. Scale bars = 20 μm. Three independent experiments were repeated with similar results. (f) Generation of *rr2b* knockout alleles in the c.v. W82 background by CRISPR–Cas9 gene editing. Two different edited events, *rr2b-1* (–1 bp) and *rr2b-2* (+1 bp), are shown for homozygous plants. (g) *RR2b* expression levels in transgenic over-expression lines OE 4 and OE 14 generated in the c.v. W82 background. Expression levels were normalized to *ELF1b* and data are presented as means ± SD from three biological replicates. Different letters indicate statistically significant differences at $p < 0.05$ by one-way ANOVA analysis with Tukey's test.

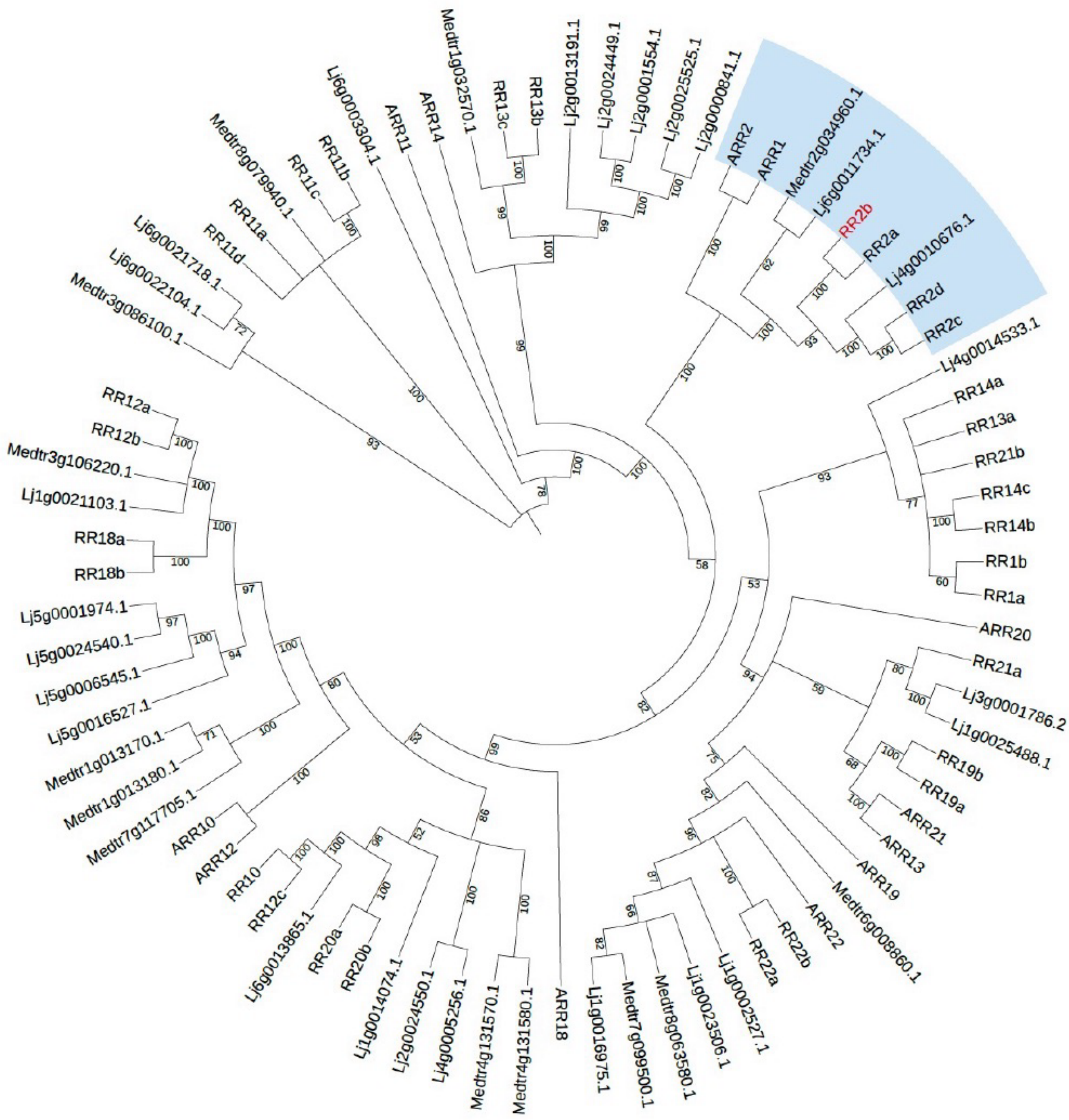

Figure S4. Phylogenetic tree of RR2b and its homologs.

The phylogenetic tree of RR2b proteins from *A. thaliana* (At), *G. max* (Gm), *L. japonicus* (Lj) and *M. truncatula* (Mt) was constructed using MEGA12. The numbers on the branches denote bootstrap values, representing the reliability of each branch.

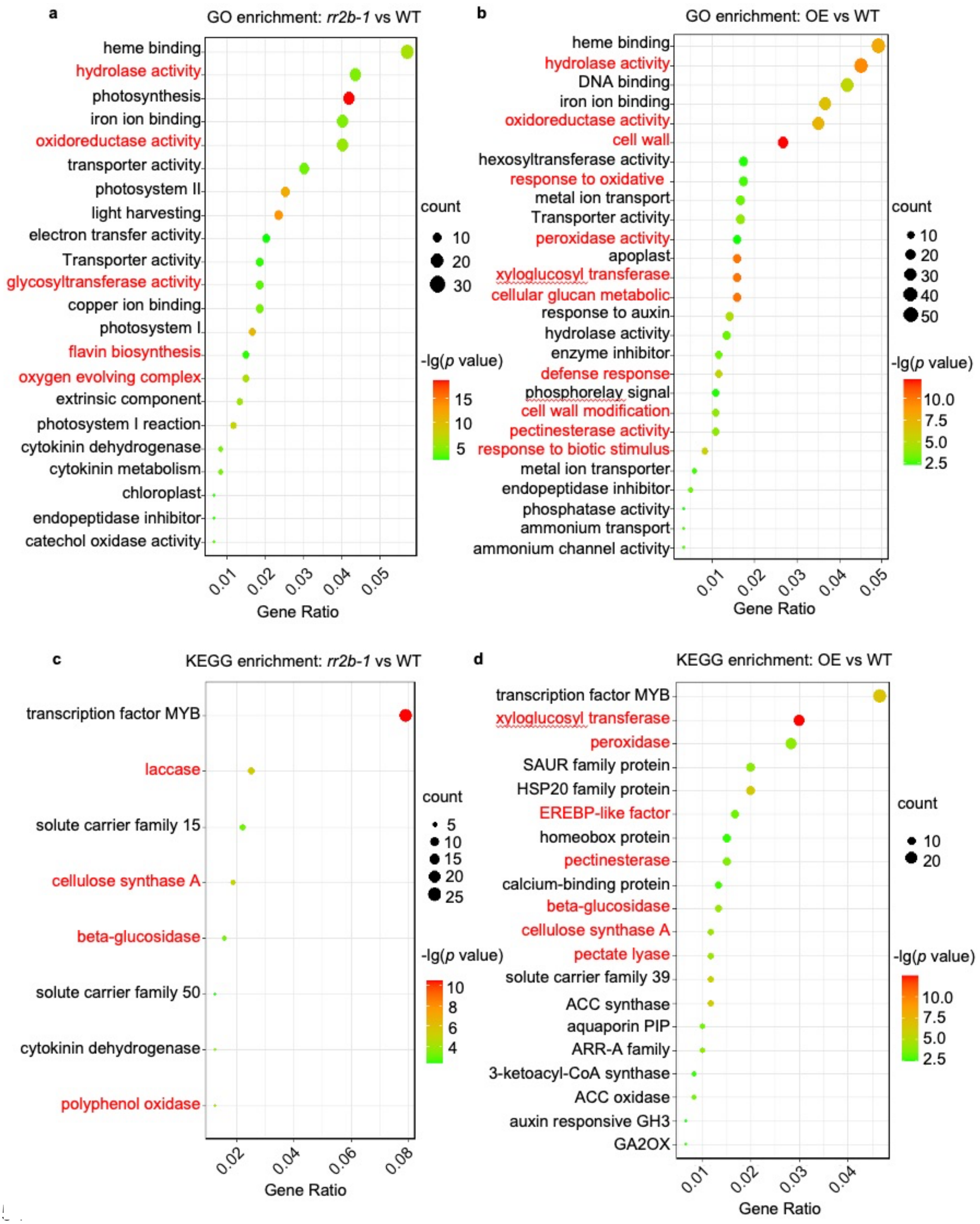

Figure S5. Cell-wall-biosynthesis and plant–pathogen-interaction-related pathways are enriched in *rr2b* knockout and *RR2b* over-expression backgrounds.

DEGs were identified from raw RNA-seq count matrix using DESeq2 based on

pairwise comparisons between *rr2b-1* and WT or OE and WT samples with no rhizobium inoculation. Gene Ontology (GO) and KEGG pathway enrichment analyses were then performed separately for each comparison. For GO enrichment analysis, soybean genes were matched to the annotation file downloaded from Phytozome (https://phytozome-next.jgi.doe.gov/) to construct a gene-to-GO table, and GO term descriptions were retrieved using the R package GO.db. GO enrichment was performed using over-representation analysis (ORA), and terms with p-values < 0.05 were considered as significantly enriched. For KEGG analysis, soybean genes were first assigned to KEGG Orthology (KO) terms based on the same annotation file, then KO terms were mapped to KEGG pathways using the R package KEGGREST to generate a gene-to-pathway annotation set. KEGG pathway enrichment was performed using the enricher function in the clusterProfiler package based on the hypergeometric test. The tested gene set in each corresponding RNA-seq comparison was used as the background, and Benjamini–Hochberg testing was applied to correct for multiple hypothesis testing. Pathways with p-values < 0.05 and q-values < 0.05 were regarded as significantly enriched. Results were visualized using ggplot2, with panels (a) and (b) showing GO enrichment for *rr2b-1* vs WT and OE vs WT, and panels (c) an (d) showing KEGG pathway enrichment for *rr2b-1* vs WT and OE vs WT, respectively.

Red font denotes cell-wall-biosynthesis and plant–pathogen-interaction-related pathways.

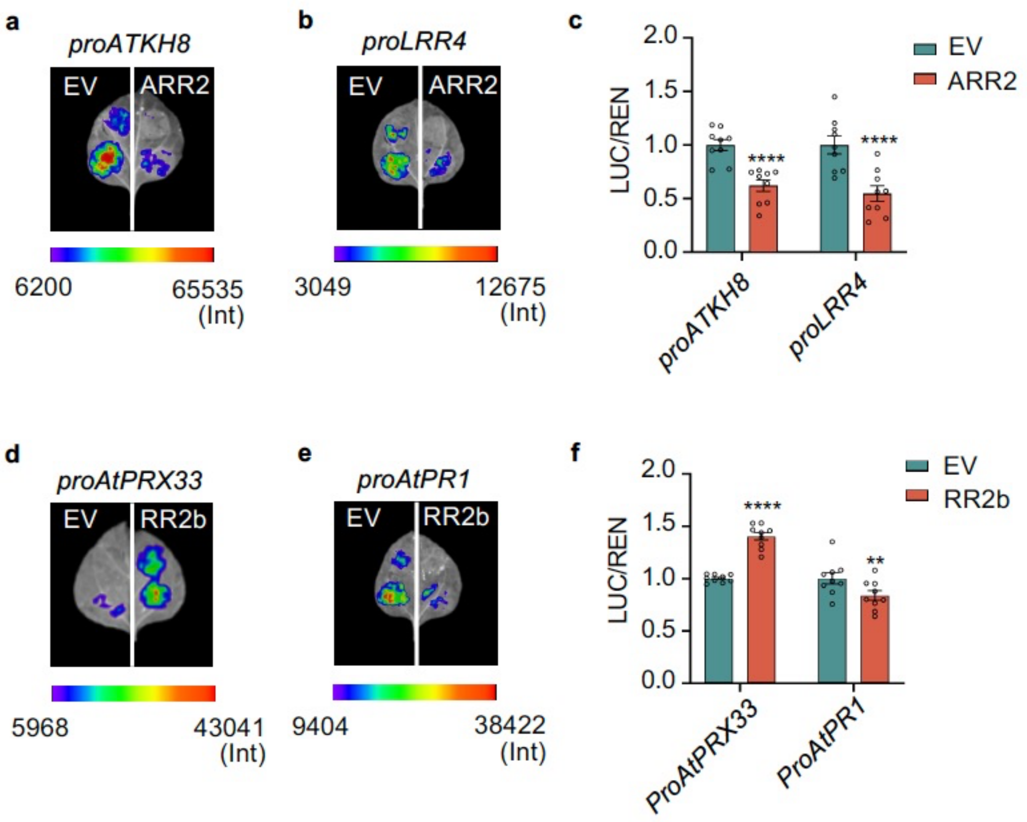

Figure S6. The effects of Arabidopsis ARR2 and soybean RR2b on disease and ROS marker genes.

(a–b) Transient dual-luciferase assays of ARR2 binding to the promoter of soybean *ATKH* (a) and *LRR4* (b). (c) Quantification of panels (a) and (b). (d–e) Transient dual-luciferase assays of RR2b binding to the promoter of *AtPRX33* (d) and *AtPR1* (e). (f) Quantification of panels (d) and (e). Shown are relative ratios of the transcriptional activities conferred by ARR2 and RR2b expression to the empty-vector (EV) control, respectively. LUC/REN, ratio of firefly luciferase (LUC) to *Renilla* luciferase (REN) activity. Data are means ± SD (n = 9). Three independent experiments were repeated with similar results. Asterisks indicate statistically significant differences relative to the EV control. Two-sided Student's t-test, **$p < 0.01$, ****$p < 0.0001$.

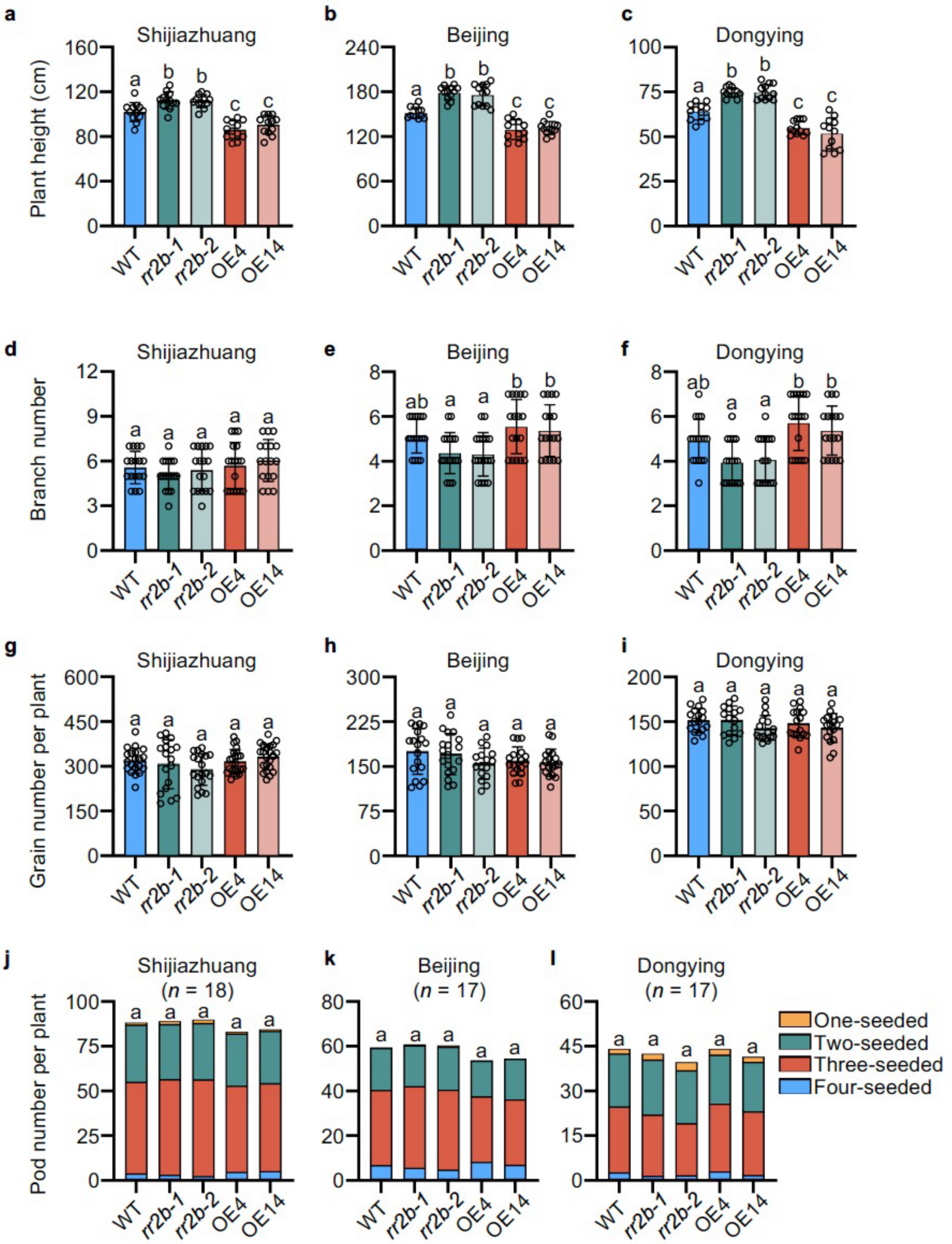

Figure S7. Over-expression and knockout of *RR2b* in soybean alter plant height but do not affect branch number, grain number per pod, and pod number in the field.

(a–c) Plant height for W82, *rr2b* knockout and *RR2b* OE lines grown at Shijiazhuang (a), Beijing (b) and Dongying (c) field sites in 2024. (d–f) Branch numbers of W82, *rr2b* knockout and *RR2b* OE lines grown at multiple field sites

in 2024. (g–i) Grain number per plant of W82, *rr2b* knockout and *RR2b* OE lines grown at multiple field sites in 2024. (j–l) Pod number per plant of W82, *rr2b* knockout and *RR2b* OE lines grown at multiple field sites in 2024. For a–i, data are presented as means ± SD (n = 18 individual plants) and different letters indicate statistically significant differences in a one-way ANOVA with Tukey’s test ($p < 0.05$). For j–l, the number of biological replicates is indicated on the graph. Significance was analyzed by Fisher's exact test compared with W82 ($p < 0.05$).

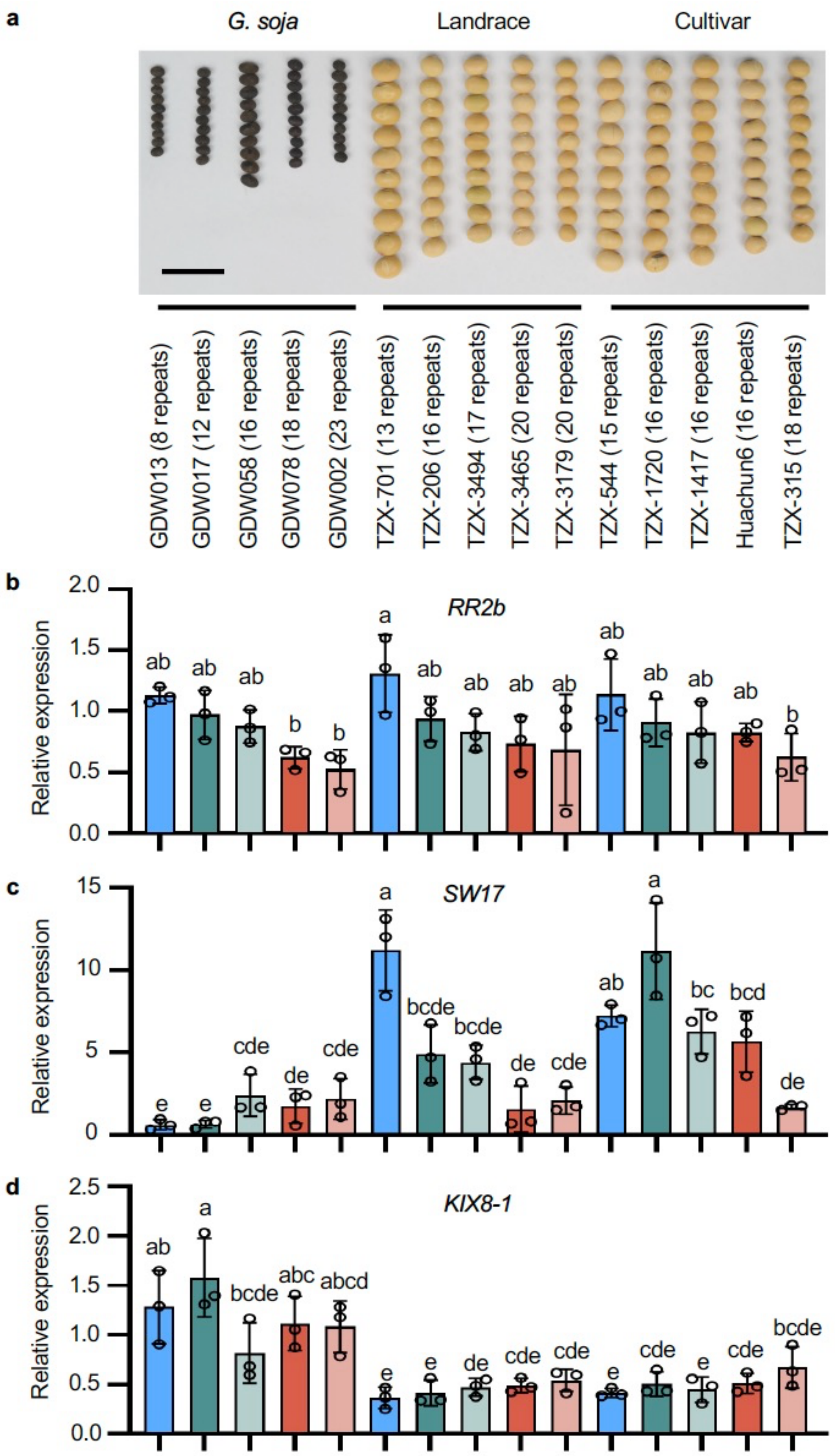

Figure S8. Expression levels of seed size-regulation genes in soybean germplasms.

(a) Seed size for 15 germplasms. Scale bars = 2 cm. (b–d) Relative expression

of *RR2b* (b), *SW17* (c) and *KIX8-1* (d) in the 15 germplasms. The soybean germplasm represented by each bar in panels b–d and the number of ATT repeats in their *RR2b* promoters are indicated below panel a. Data are presented as means ± SD from three biological replicates and different letters indicate statistically significant differences at $p < 0.05$ by one-way ANOVA with Tukey’s test.

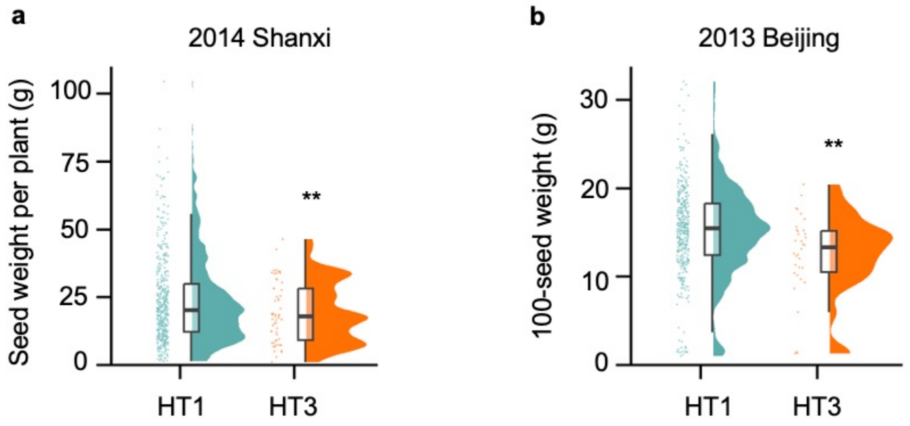

Figure S9. Hundred-seed weight and seed-weight per plant of *G. max* accessions of different *RR2b* haplotypes.

(a) The seed-weight per plant for HT1 (n = 463) and HT3 (n = 56) grown in field in Shanxi in 2014. (b) Hundred-seed weight for HT1 (n = 396) and HT3 (n = 31) grown in field in Beijing in 2013. Data were collected from SoyOmics database and are presented as means ± SD. Asterisks indicate statistically significant differences relative to HT1. Two-sided Student's t-test and Wilcoxon rank-sum test, $^{**}p < 0.01$.

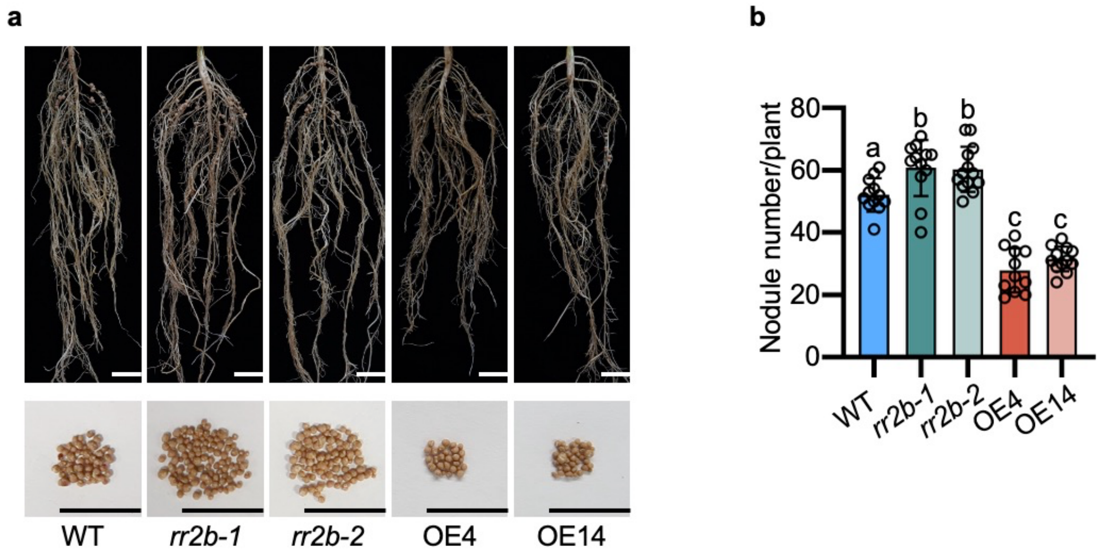

Figure S10. RR2b is a negative regulator of soybean nodulation.

(a) Nodule phenotype for W82, *rr2b* knockout and *RR2b* OE lines. (b) Nodule numbers per plant for W82, *rr2b* and *RR2b* OE plants. Data are means ± SD (n = 18 individual plants). Three independent experiments were performed with similar results.

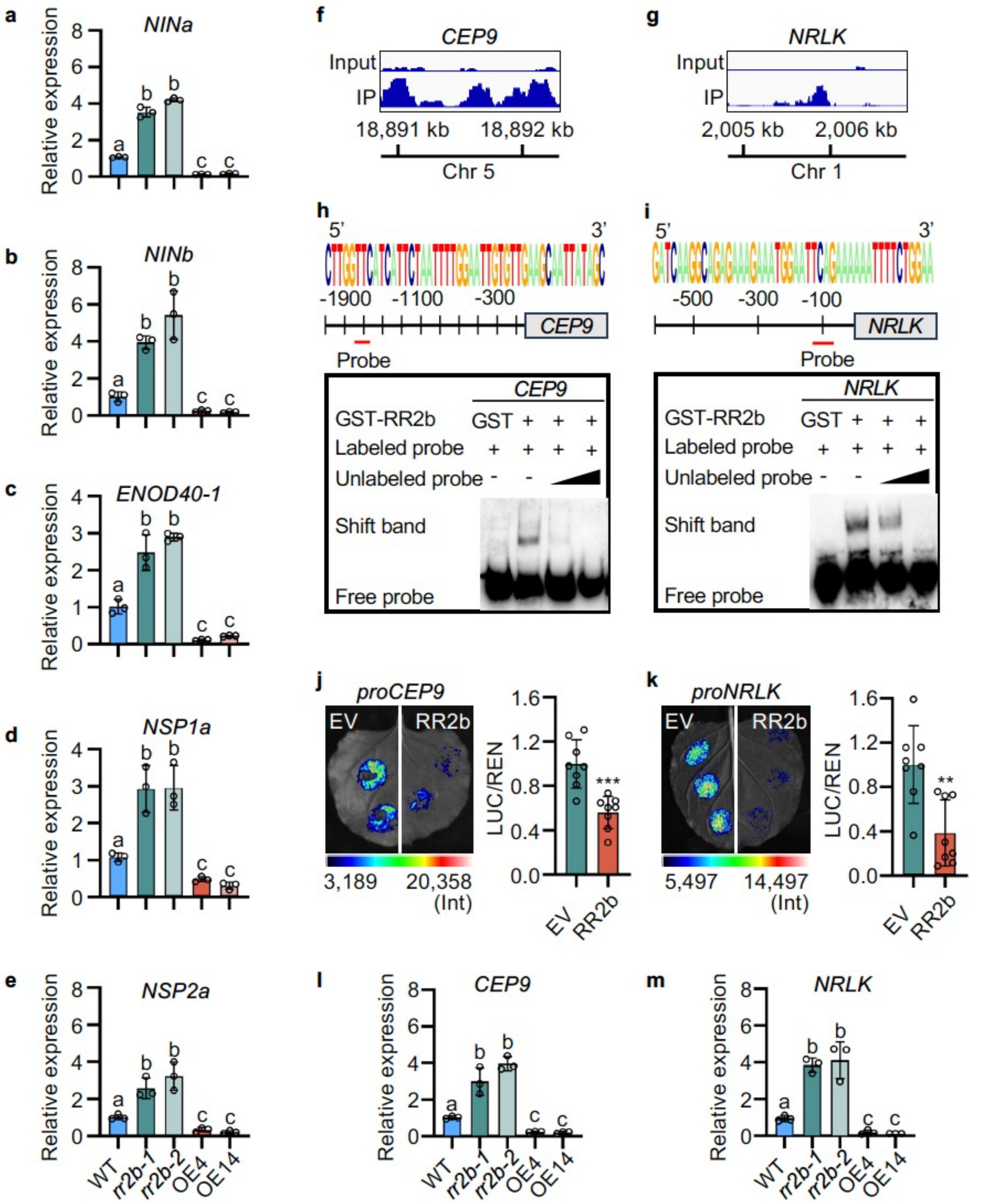

Figure S11. RR2b represses major soybean nodulation-pathway genes.

(a–e) Relative expression of nodulation-pathway marker genes, *NINa* (a), *NINb* (b), *ENOD40-1* (c), *NSP1a* (d) and *NSP2a* (e) in W82, *rr2b* knockout and *RR2b* OE lines. Data are presented as means ± SD from three biological replicates. (f, g) CUT & Tag analysis of RR2b preferentially binding to the promoters of *CEP9* (f) and *NRLK* (g). (h, i) EMSA of GST–RR2b binding *in vitro* to *cis*-

elements in the promoters of *CEP9* (h) and *NRLK* (i). The colored nucleotide sequence at the top of each panel represents the probe sequence. The red lines indicate the exact location of the probe within the promoter region. Three independent replicates were performed and a representative result is shown. (j, k) Transient dual-luciferase assays of RR2b binding to the promoters of *CEP9* (j) and *NRLK* (k). Data are presented as means ± SD ($n = 8$). Three independent experiments were repeated with similar results. Asterisks indicate statistically significant differences relative to the EV control. Two-sided Student's *t*-test, $^{**}p < 0.01$, $^{***}p < 0.001$. (l, m) Relative expression of *CEP9* (l) and *NRLK* (m) in W82, *rr2b* knockout and *RR2b* OE lines. Data are means ± SD from three biological replicates.

In a–e, l and m, expression levels were normalized to *ELF1b*. Different letters indicate statistically significant differences at $p < 0.05$ by one-way ANOVA analysis with Tukey's test.

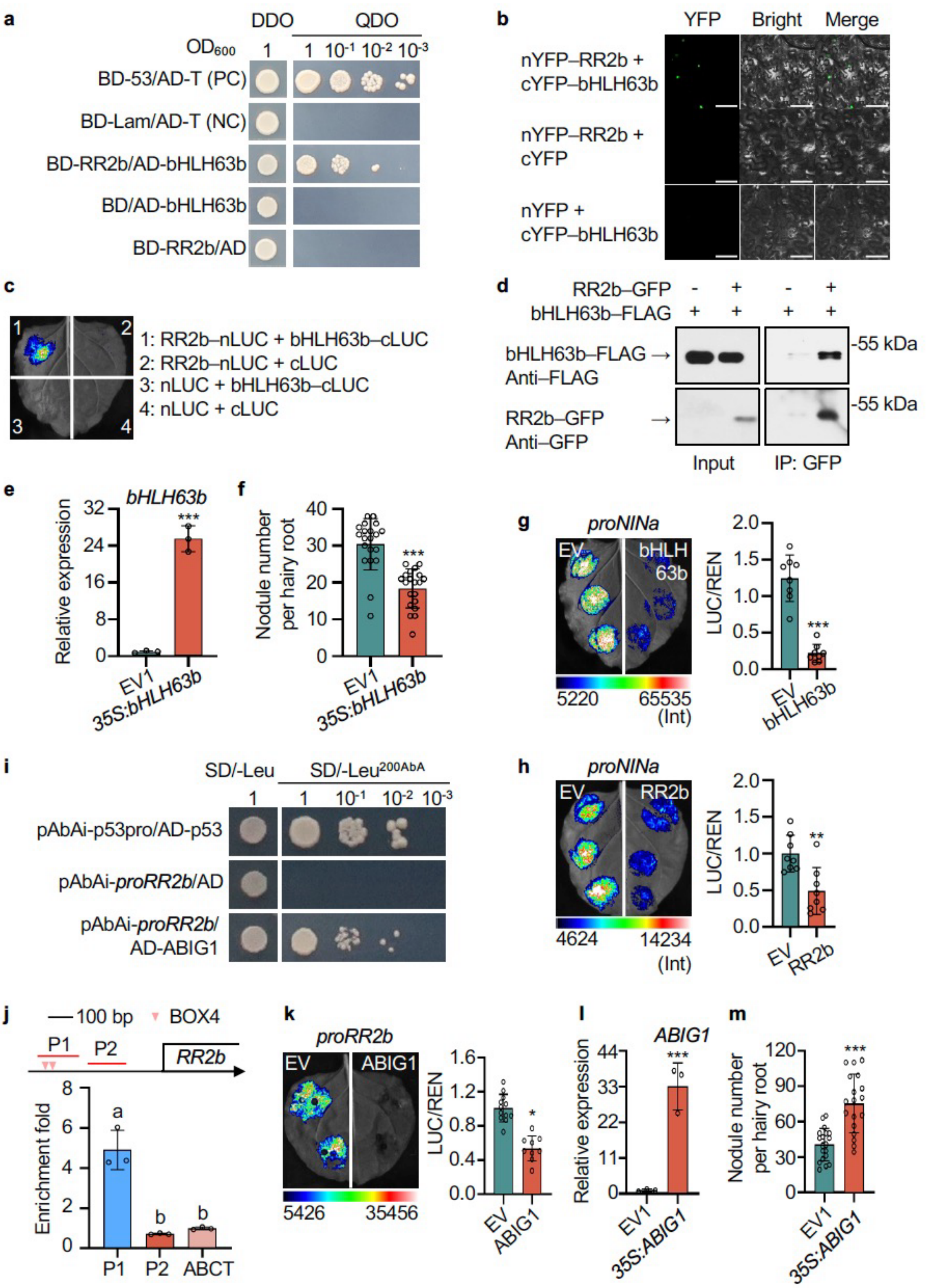

Figure S12. The *ABIG1–RR2b–bHLH63* genetic pathway regulates soybean nodulation.

(a) Yeast two–hybrid assay of the physical interaction between RR2b and bHLH63b. Cells were grown on DDO (–Trp/–Leu) or QDO (–Trp/–Leu/–His/–Ade) synthetic dropout medium and numbers at the top indicate four serial

dilutions. AD, GAL4 activation domain; BD, GAL4 DNA-binding domain. (b) Bi-molecular fluorescence complementation analysis of the physical interaction between RR2b and bHLH63b in transiently transgenic *N. benthamiana* leaves. Scale bars, 100 μm. cYFP, C-terminal portion of YFP. nYFP, N-terminal portion of YFP. (c) Luciferase-complementation imaging assay in transiently transgenic *N. benthamiana* leaves testing the interaction between RR2b and bHLH63b. Representative images of *N. benthamiana* leaves at 48 h after infiltration are shown. (d) Co-IP analysis of the physical interactions between RR2b-GFP and bHLH63b–3xFLAG in soybean hairy roots. (e) *bHLH63b* expression level in transgenic hairy roots expressing $35S_{pro}$:*bHLH63b* or an empty-vector control (EV1). (f) Quantification of nodule number per hairy root expressing EV1 and $35S_{pro}$:*bHLH63b*. Data are means ± SD of 20 hairy roots per construct. (g) Transient dual-luciferase assays of bHLH63b binding to the promoter of *NINa*. Shown are relative ratios of the transcriptional activities conferred by bHLH63b expression to the empty vector control. (h) Transient dual-luciferase assays of RR2b binding to the promoter of *NINa*. (i) Yeast one-hybrid assay of ABIG1 binding to the *RR2b* promoter. Numbers along the top indicate four serial dilutions. (j) ChIP-qPCR assay of ABIG1 binding to the promoter of *RR2b*. Red lines indicate fragments amplified for ChIP-qPCR, and inverted triangles represent BOX4 motifs (ATTAAT) bound by ABIG1. (k) Transient dual-luciferase assays of ABIG1 binding to the promoter of *RR2b*. (l) Relative *ABIG1* expression in transgenic hairy roots expressing $35S_{pro}$:*ABIG1* or an empty–vector control (EV1). (m) Quantification of nodule number per hairy root expressing EV1 and $35S_{pro}$:*ABIG1* constructs. Data are means ± SD (n = 19).

In b–g and h, k–m, three independent experiments were repeated with similar results. In g, h, k, LUC/REN, ratio of firefly luciferase to *Renilla* luciferase activity. Data are presented as means ± SD (n = 8). Three independent experiments were repeated with similar results. Asterisks indicate statistically significant differences relative to the EV control. Two-sided Student's t-test, ***p < 0.001,

**$p < 0.01$, *$p < 0.05$. In e and l, gene-expression levels were normalized to *ELF1b* and data are means ± SD from three biological replicates. In panels e, f, l, m, asterisks indicate statistically significant differences relative to the EV1 control. Two-sided Student's *t*-test, ***$p < 0.001$. In panel j, two independent experiments were repeated with similar results. Data are presented as means ± SD (n = 3). Different letters indicate statistically significant differences in a one-way ANOVA analysis with Tukey's test ($p < 0.05$).

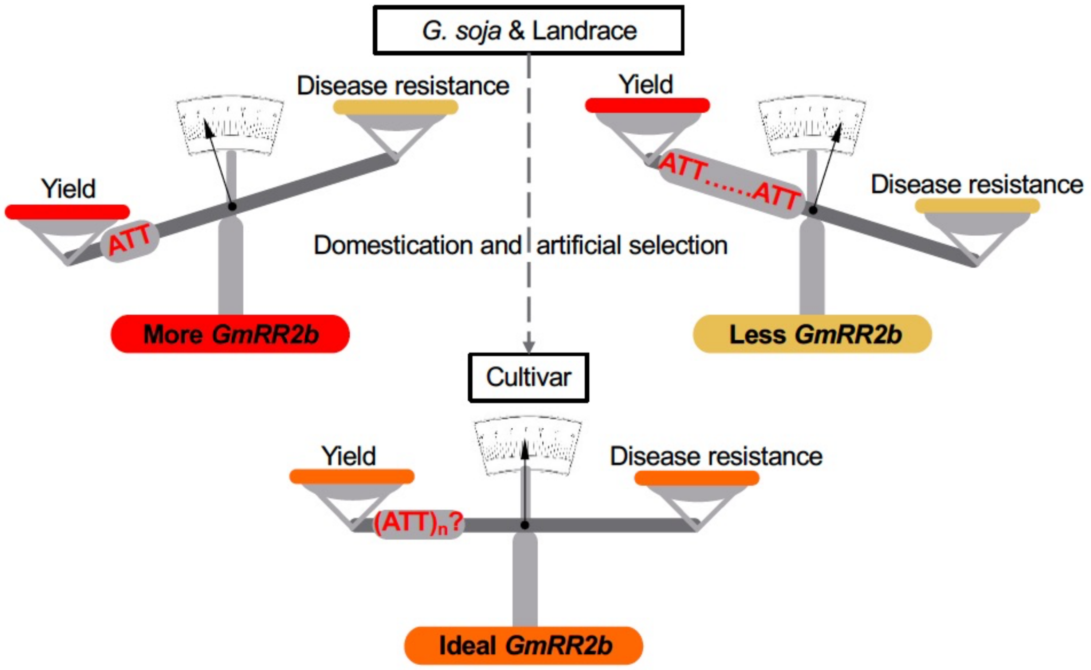

Figure S13. Proposed working model of RR2b function in balancing yield and disease resistance in soybean.

In both wild soybeans and landraces, the *RR2b* promoter has varying lengths of ATT insertion fragments. Generally, longer insertion fragments correlate with weaker promoter activity and enhanced disease resistance in the corresponding varieties, but at the cost of reduced yield (the top-right balance). Conversely, shorter fragments tend to enhance yield but diminish disease resistance (the top-left balance). Throughout the soybean domestication process, haplotypes with moderate insertion–fragment lengths that facilitate optimal *RR2b* promoter activity were preserved, achieving a balance between yield and disease resistance (the bottom balance). The color of the balance pan indicates relative intensity levels for *RR2b* expression: red signifies high intensity, yellow indicates low intensity, and orange denotes moderate intensity. The (ATT) insertion fragment represents the rider of the balance, precisely regulating soybean yield and disease resistance. The uncertain n and the question mark denote that the optimal *RR2b* activity, as determined by the

length of the insertion fragment in its promoter, may vary under different environmental conditions for achieving an optimal yield-disease resistance balance in soybeans.

References

76. Wu, X. et al. SNP discovery by high-throughput sequencing in soybean. *BMC Genomics* **11**, 469 (2010).
77. Abdelmajid, K. M. et al. Quantitative trait loci (QTL) that underlie SCN resistance in soybean [*Glycine max* (L.) Merr.] PI438489B by 'Hamilton' re-combinant inbred line (RIL) population. *Atlas J. Plant Biol.* **1**, 29-38 (2014).
78. Ning, H. et al. Identification of QTLs related to the vertical distribution and seed-set of pod number in soybean *Glycine max* (L.) Merri. *PLoS One* **13**, e0195830. (2018).
79. Luckew, A. S., Swaminathan, S., Leandro, L. F., Orf, J. H. & Cianzio, S. R. 'MN1606SP' by 'Spencer' filial soybean population reveals novel quantitative trait loci and interactions among loci conditioning SDS resistance. *Theor. Appl. Genet.* **130**, 2139-2149 (2017).
80. Harris, D. K. et al. Soybean quantitative trait loci conditioning soybean rust-induced canopy damage. *Crop Sci.* **55**, 2589-2597 (2015).
81. Zhang, D. et al. Identification of genomic regions determining flower and pod numbers development in soybean (*Glycine max* L.). *J. Genet. Genomics* **37**, 545-556 (2010).
82. Wang, Y. et al. Mapping isoflavone QTL with main, epistatic and QTL x environment effects in recombinant inbred lines of soybean. *PLoS One* **10**, e0118447 (2015).
83. Han, Y. et al. Unconditional and conditional QTL underlying the genetic interrelationships between soybean seed isoflavone, and protein or oil contents. *Plant Breed.* **134**, 300-309 (2015).
